# Programmable Multidimensional Diffraction Engineering via an Optically Addressed Nested Fourier Metasurface

**DOI:** 10.1002/nap2.70309

**Published:** 2026-10-07

**Authors:** Jing Guo, Qinglei Zhao, Jingrui Yang, Zongyi Liu, Mingdong Zhu, Shuai Liu, Chao Li, Shuxin Wang, Qinglong Hu, Fengwei Guan, Qiang Liu, Qi Song

**Affiliations:** ^1^ Changchun Institute of Optics, Fine Mechanics and Physics Chinese Academy of Sciences Changchun China; ^2^ University of Chinese Academy of Sciences Beijing China; ^3^ Yuelushan Laboratory Changsha China; ^4^ State Key Laboratory of Hybrid Rice Hunan Academy of Agricultural Sciences Changsha China; ^5^ Qualcomm Inc. San Diego California USA; ^6^ Soleilware Photonics LLC. San Diego California USA

**Keywords:** Fourier optimization, metasurface, optical routing, polarization multiplexing

## Abstract

Free‐space optical routing and programmable Fourier optical processing are promising for next‐generation photonic information systems. Here, we propose an optically addressed nested Fourier metasurface (OA‐NFM) platform for dynamically switchable, multidimensional optical routing and diffraction‐channel engineering without dense electrical interconnects. By integrating a Fourier‐coefficient‐based inverse‐design method with a nested Fourier metasurface architecture, the proposed platform enables simultaneous control of diffraction channels and polarization states. By spatially multiplexing Fourier‐engineered mode regions within nested supercells and activating them through digital light processing (DLP)‐based optical addressing, the OA‐NFM achieves programmable momentum‐space redistribution, sideband clustering, and angular‐channel engineering via controllable superlattice diffraction. The local Fourier mode defines the target diffraction response, while the global nested array factor controls the angular distribution of clustered sidebands. Simulations show that individually optimized modes achieve diffraction efficiencies up to 45% with polarization fidelities above 0.99. The OA‐NFM achieves diffraction efficiencies up to 37% within the ± 1.1° receiver window, excluding losses from the DMD and projection optics. NRZ‐OOK simulations confirm BERs below the hard‐decision FEC threshold (10^−3^) at 25 Gbps across the 1500–1600 nm band. This work demonstrates a promising platform for optical switching, free‐space routing, and multidimensional photonic information processing.

## Graphical abstract

An optically addressed nested Fourier metasurface is presented for programmable multidimensional diffraction routing without electrical interconnects. Local Fourier‐mode engineering combined with global superlattice diffraction enables on‐demand reconfigurable routing. System‐level simulations demonstrate 25 Gbps NRZ‐OOK transmission with open eye diagrams, highlighting a promising route toward high‐speed, reconfigurable free‐space optical interconnects.

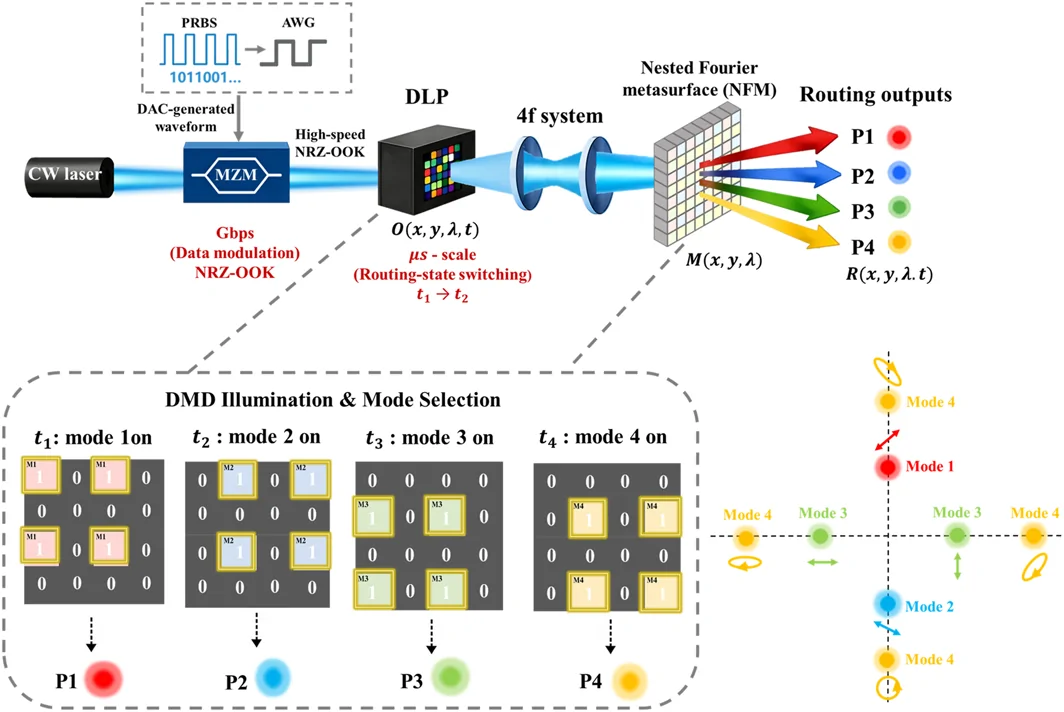

## 1 Introduction

The rapid development of artificial intelligence, high‐performance computing, and photonic information processing has created a growing demand for compact and programmable platforms capable of manipulating multiple optical degrees of freedom [1, 2]. Beyond conventional optical communication and data‐center interconnects, dynamic wavefront engineering is becoming increasingly important for diverse applications including Fourier‐domain optical processing, holography, and free‐space optical interconnects. Consequently, developing scalable photonic architectures that enable dynamic manipulation of multidimensional optical degrees of freedom on a unified hardware platform has become a central challenge.

Conventional optical switching and beam‐routing technologies generally involve a trade‐off between scalability and speed. Highly scalable approaches, such as micro‐electromechanical systems (MEMS), liquid‐crystal‐on‐silicon (LCoS), and thermo‐optic switches, provide large port counts but are limited by millisecond‐level response times [3]. In contrast, fast photonic switches based on electro‐optic Mach–Zehnder interferometers (MZIs), semiconductor optical amplifiers (SOAs), and arrayed waveguide gratings (AWGRs) can provide nanosecond‐scale switching, but often suffer from high wiring complexity, large insertion loss, poor scalability, and high power consumption [4, 5, 6, 7, 8]. More fundamentally, these architectures are primarily optimized for routing between predefined ports rather than for simultaneous control of diffraction channels, polarization states, and other optical degrees of freedom.

Metasurfaces, composed of subwavelength meta‐atoms arranged in two‐dimensional arrays, provide a compact platform for flexible optical‐field manipulation [9, 10, 11, 12]. By tailoring the amplitude, phase, polarization, and momentum‐space response of individual meta‐atoms, they enable diverse functionalities, including directive radiation and Fourier‐space manipulation, within deeply subwavelength thicknesses. These capabilities have been widely exploited for beam steering, holography, optical multiplexing, polarization manipulation, and Fourier optical processing [13, 14, 15, 16, 17, 18, 19, 20]. However, dynamic metasurfaces commonly rely on electrically driven mechanisms, such as phase‐change materials, liquid crystals, carrier injection, and thermo‐optic modulation [21]. Scaling these approaches to large‐area or high‐density arrays requires increasingly complex electrical interconnects.

Optically addressed metasurfaces constitute an attractive platform for dynamic, spatially‐resolved wavefront control. External optical illumination can directly tune local optical responses, eliminating the dense, complex electrical interconnects mandatory for electrically addressed architectures [22, 23, 24]. Recent advances have enabled pixel‐level and even finer‐scale optical programming for beam steering, wavefront shaping, and spatial light modulation. However, these studies have primarily focused on the dynamic programmability of local wavefront responses. Extending such approaches toward multichannel optical communications and routing imposes more stringent requirements beyond local reconfigurability, including high diffraction efficiency, strong channel isolation, polarization selectivity, and scalable channel multiplexing. In particular, increasingly fine spatial addressing may involve trade‐offs in optical power utilization and effective diffraction efficiency. More importantly, it remains a major challenge to efficiently redistribute incident optical power across multiple predefined spatial, polarization‐selective diffraction channels while suppressing inter‐channel crosstalk and preserving reliable system‐level transmission performance.

By combining Fourier‐coefficient‐driven design with digital‐light‐processing (DLP) optical addressing, the metasurface enables dynamic selection of multiple spatial and polarization diffraction channels without dense electrical wiring. We further introduce a nested Fourier metasurface (NFM) supercell architecture, in which the interplay between locally engineered Fourier modes and the global array factor governs momentum redistribution and angular sideband formation. This hierarchical diffraction mechanism enables programmable angular‐channel control while preserving the target diffraction functionality encoded by the local metasurface phase profile.

As a proof of concept, the generated diffraction sidebands are collected within the receiver angular window to enhance the useful power for free‐space optical communication, and the transmission performance is evaluated through eye diagrams and bit‐error‐rate (BER) analysis under non‐return‐to‐zero on‐off keying (NRZ‐OOK) modulation. By linking programmable diffraction engineering with communication‐level performance, the proposed platform provides a compact and reconfigurable approach for multidimensional optical routing, photonic interconnects, and free‐space communications.

The remainder of this paper is structured as follows. Section 2 outlines the operating principle and design framework of the optically addressed nested Fourier metasurface platform. Section 3 presents the device‐level optimization and simulation results from free‐space optical communication at the system level. Section 4 discusses the underlying physical mechanisms and explores their broader implications. Section 5 concludes the work with a summary of key findings.

## 2 Device Concept and Design

### 2.1 System Architecture and Optical Addressing Principle

The OA‐NFM platform integrates programmable optical addressing with a static nested Fourier metasurface to realize dynamically multidimensional diffraction routing, as illustrated in Figure 1a. A DLP module incorporating a digital micromirror device (DMD) serves as the optical addressing layer. The Mach–Zehnder modulator (MZM) carries the high‐speed NRZ‐OOK data, whereas the DMD provides quasi‐static routing‐state selection, projecting programmable binary illumination patterns onto the metasurface plane through a 4f imaging system. At the core of the platform lies a nested Fourier metasurface composed of a periodic array of nested supercells. Each nested supercell comprises a 2 × 2 arrangement of four independently designed mode regions (Modes 1–4), each engineered to generate a prescribed combination of diffraction order and polarization state. As illustrated in Figure 1b, dynamically updating the DMD projection pattern selectively activates different mode regions, routing the incident beam to the corresponding output ports (P1–P4) with their encoded polarization characteristics. Figure 1c depicts the momentum‐space distributions of the four modes, showing their distinct angular and polarization routing states. Thus, multidimensional routing states can be reconfigured entirely through the external illumination pattern without modifying the metasurface nanostructure.

**FIGURE 1:**
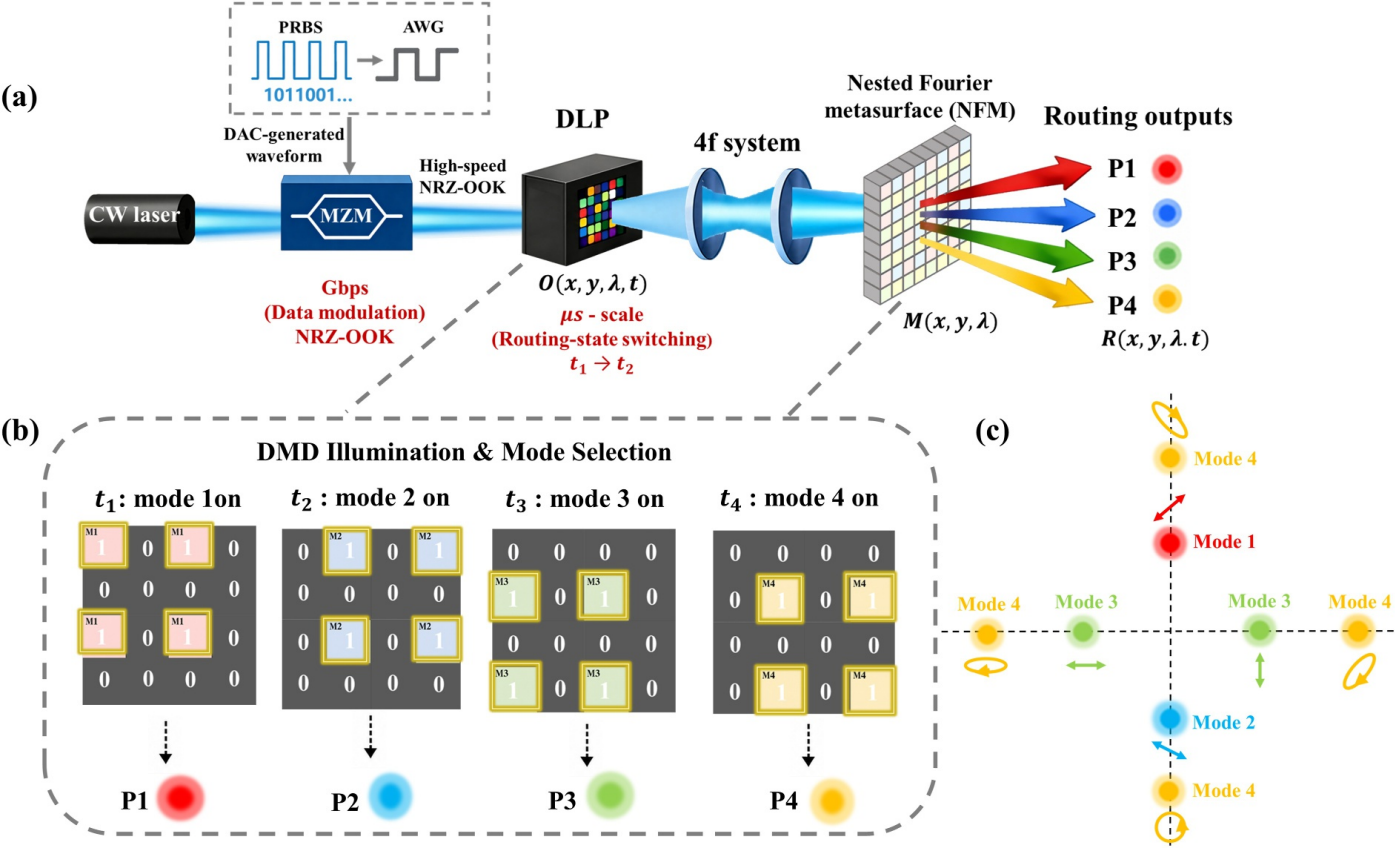
System architecture and optical addressing principle of the OA‐NFM routing platform. (a) Schematic of the OA‐NFM routing platform. A continuous‐wave (CW) laser is modulated by an MZM and projected onto the nested Fourier metasurface through a 4f imaging system for dynamic routing to outputs (P1–P4). (b) Illustration of DMD‐based optical addressing: different mode regions (Modes 1–4) are selectively activated, demonstrating programmable switching among multiple outputs. (c) Momentum‐space representation of multimode diffraction routing for Modes 1–4.

The projected optical field acts as a spatially selective gating signal. This binary addressing logic is captured by the modulation function:

(1) $$\begin{array}{c} O\left(x,y,\lambda ,t\right)=\left\{\begin{array}{ll} 0, & \text{inactive}\;\text{state}\\ 1, & \text{active}\;\text{state} \end{array}\right. \end{array}.$$

When *O* = 1, the corresponding mode region is illuminated and its encoded diffraction channel is excited. When *O* = 0, the reflected field is deflected away from the working optical path.

At the system level, the overall optical response can therefore be modeled as the product of the static metasurface transmission and the programmable addressing function:

(2) $$\begin{array}{c} R\left(x,y,\lambda ,t\right)=O\left(x,y,\lambda ,t\right)M\left(x,y,\lambda \right), \end{array}$$

where M(x,y,λ) is the metasurface response and O(x,y,λ,t) corresponds to the spatiotemporal optical addressing function generated by the DMD. In the nested‐supercell architecture, each addressing unit maps to an entire mode region rather than a single meta‐atom. The effective system response thus takes the form of a mode‐selective superposition:

(3) $$\begin{array}{c} R_{\text{eff}}\left(x,y,\lambda ,t\right)=\sum_{i=1}^{4}O_{i}\left(x,y,\lambda ,t\right)\;M_{i}\left(x,y,\lambda \right), \end{array}$$

where *M*_*i*_ denotes the metasurface response associated with the *i*th mode region, and *O*_*i*_ represents the corresponding optical‐addressing mask function. By tailoring the spatial distribution of $\sum_{i=1}^{4}O_{i}\left(x,y,\lambda ,t\right)$, programmable multichannel diffraction routing and mode‐selective output can be realized within the same planar device platform.

The overall diffraction‐routing reconfiguration speed of the OA‐NFM system is primarily governed by the DMD refresh rate, yielding a typical channel‐switching time of *T*_switch_ ≈ 10–100 μs [25, 26]. This enables rapid switching among the pre‐engineered diffraction‐routing channels through external optical addressing, without modifying the metasurface structure.

### 2.2 Nested Fourier Metasurface Design Framework

As shown in Figure 2, the nested supercell employs a hierarchical spatially multiplexed architecture, consisting of a 2 × 2 arrangement of mode regions. Each mode region further integrates an 8 × 8 array of identical mode super‐pixels. The proposed layout offers two key advantages: first, it enables spatial multiplexing of the four diffraction‐polarization modes within a single aperture; second, the enlarged repetition count *N* reduces the angular separation between adjacent superlattice sidebands, resulting in a denser distribution of these sidebands around the main diffraction peak. This denser sideband distribution improves the effective energy utilization and enhances compatibility with the angular acceptance bandwidth of the downstream detection optics. Each mode super‐pixel is composed of a 10 × 10 array of asymmetric rectangular nanopillars. Inverse Fourier‐coefficient optimization determines their in‐plane dimensions to encode the target diffraction order and polarization state, providing the structural basis for optically addressed multichannel routing.

**FIGURE 2:**
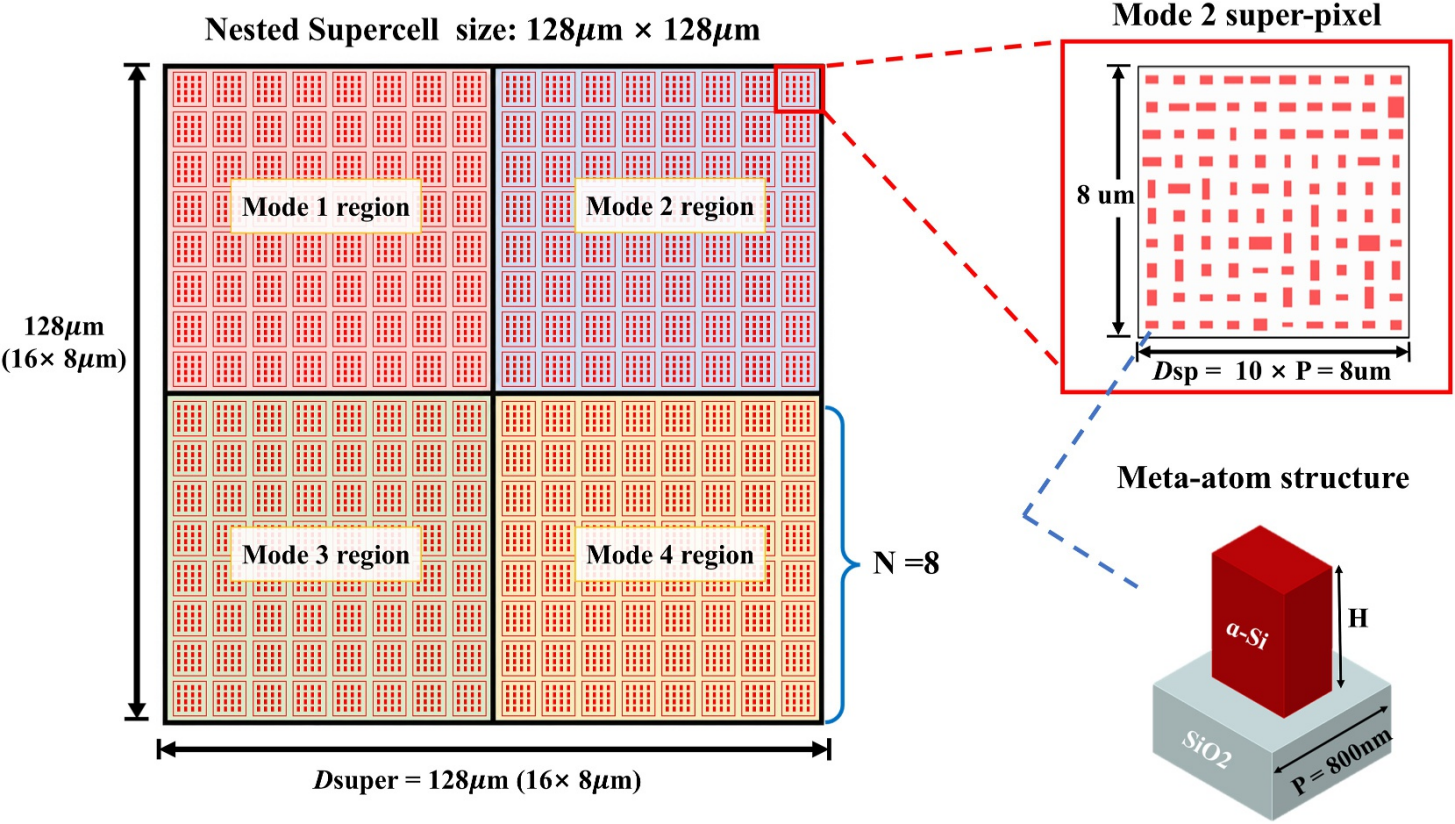
Nested supercell design of the NFM. Schematic of the NFM routing configuration featuring a 128 μm × 128 μm nested supercell (2 × 2 arrangement, Modes 1–4 regions) and meta‐atom period *P* = 800 nm.

#### 2.2.1 Fourier‐Coefficient‐Based Inverse Design of Super‐Pixels

To achieve simultaneous control over diffraction orders and polarization states, we employ a Fourier‐coefficient‐based optimization framework to independently engineer four target diffraction modes [27, 28]. Unlike conventional approaches based on phase superposition or geometric‐phase modulation, the proposed method maps diffraction efficiency and polarization information onto a unified complex‐vector Fourier‐coefficient representation. This formulation enables concurrent manipulation of multiple diffraction channels and their associated polarization degrees of freedom within a single optimization framework.

Each mode super‐pixel consists of a 10 × 10 array of meta‐atoms with a lattice period of *P* = 800 nm, corresponding to a super‐pixel period *D* = *D*sp = 10 × *P* = 8 μm. The local optical response of a super‐pixel is described by the spatially varying Jones transmission matrix [29, 30, 31]:

(4) $$\begin{array}{c} T\left(x_{p},y_{p}\right)=\left(\begin{array}{cc} t_{xx}e^{i\Phi_{xx}\left(x_{p},y_{p}\right)} & t_{xy}e^{i\Phi_{xy}\left(x_{p},y_{p}\right)}\\ t_{yx}e^{i\Phi_{yx}\left(x_{p},y_{p}\right)} & t_{yy}e^{i\Phi_{yy}\left(x_{p},y_{p}\right)} \end{array}\right), \end{array}$$

where ($x_{p},y_{p}$) denotes the local coordinate within the super‐pixel and serves as the integration variable in the Fourier expansion, distinguishing the pixel‐level position from the global metasurface coordinate (*x*, *y*).

Owing to the symmetry of the rectangular nanopillars and the alignment of their principal axes with the *x*‐ and *y*‐axes, polarization conversion between the two orthogonal linear‐polarization eigenchannels is negligible, such that $t_{xy}$ ≈ $t_{yx}$ ≈ 0. Under the high‐transmission approximation, the Jones matrix can therefore be reduced to the phase‐only diagonal form:

(5) $$\begin{array}{c} T\left(x_{p},y_{p}\right)=\left(\begin{array}{cc} e^{i\Phi_{xx}\left(x_{p},y_{p}\right)} & 0\\ 0 & e^{i\Phi_{yy}\left(x_{p},y_{p}\right)} \end{array}\right), \end{array}$$

where $\Phi_{xx}\left(x_{p},y_{p}\right)$ and $\Phi_{yy}\left(x_{p},y_{p}\right)$ denote the phase responses associated with the *x*‐ and *y*‐polarized eigenchannels, respectively. Thus, each meta‐atom provides two phase degrees of freedom for tailoring the vectorial diffraction response.

According to Fourier diffraction theory, the complex transmission coefficients corresponding to the (*m*, *n*)th diffraction order are given by the two‐dimensional Fourier coefficients of the spatial transmission functions:

(6) $$\begin{array}{c} \begin{array}{rr} c_{xx}^{\left(m,n\right)} & =\frac{1}{D^{2}}\int_{0}^{D}\int_{0}^{D}e^{i\Phi_{xx}}e^{-i2\pi \left(mx_{p}+ny_{p}\right)/D}\;dx_{p}dy_{p}\\ c_{yy}^{\left(m,n\right)} & =\frac{1}{D^{2}}\int_{0}^{D}\int_{0}^{D}e^{i\Phi_{yy}}e^{-i2\pi \left(mx_{p}+ny_{p}\right)/D}\;dx_{p}dy_{p} \end{array}. \end{array}$$

These coefficients constitute the diagonal elements of the Fourier‐domain Jones matrix:

(7) $$\begin{array}{c} C^{\left(m,n\right)}=\left(\begin{array}{cc} c_{xx}^{\left(m,n\right)} & 0\\ 0 & c_{yy}^{\left(m,n\right)} \end{array}\right) \end{array}.$$

For an incident Jones vector $E_{\text{in}}$, the field in the (*m*, *n*)th diffraction order is given by $E^{\left(m,n\right)}=C^{\left(m,n\right)}E_{\text{in}}$. In the particular case of 45° linear polarization (equal‐amplitude excitation), this simplifies to:

(8) $$\begin{array}{c} E^{\left(m,n\right)}=\left(\begin{array}{c} E_{x}^{\left(m,n\right)}\\ E_{y}^{\left(m,n\right)} \end{array}\right)\;\propto \;\left(\begin{array}{c} c_{xx}^{\left(m,n\right)}\\ c_{yy}^{\left(m,n\right)} \end{array}\right) \end{array}.$$

Hence, the magnitude ratio and relative phase of $c_{xx}^{\left(m,n\right)}$ and $c_{yy}^{\left(m,n\right)}$ determine the polarization state of each diffraction channel. The corresponding Stokes parameters can be expressed as:

(9) $$\begin{array}{cc} S_{0}^{\left(m,n\right)} & ={\left|E_{x}^{\left(m,n\right)}\right|}^{2}+{\left|E_{y}^{\left(m,n\right)}\right|}^{2}\\ S_{1}^{\left(m,n\right)} & ={\left|E_{x}^{\left(m,n\right)}\right|}^{2}-{\left|E_{y}^{\left(m,n\right)}\right|}^{2}\\ S_{2}^{\left(m,n\right)} & =2\;\text{Re}\left[E_{x}^{\left(m,n\right)}E_{y}^{\left(m,n\right)*}\right]\\ S_{3}^{\left(m,n\right)} & =2\;\text{Im}\left[E_{x}^{\left(m,n\right)}E_{y}^{\left(m,n\right)*}\right] \end{array},$$

where $S_{0}^{\left(m,n\right)}$ represents the total intensity of the corresponding diffraction channel, whereas $S_{1}^{\left(m,n\right)}$, $S_{2}^{\left(m,n\right)}$, and $S_{3}^{\left(m,n\right)}$ characterize its polarization state. For a completely polarized field, the corresponding normalized Stokes vector can be written as

(10) $$\begin{array}{c} {\tilde{S}}^{\left(m,n\right)}=\left[\begin{array}{c} 1\\ S_{1}^{\left(m,n\right)}/S_{0}^{\left(m,n\right)}\\ \begin{array}{c} S_{2}^{\left(m,n\right)}/S_{0}^{\left(m,n\right)}\\ S_{3}^{\left(m,n\right)}/S_{0}^{\left(m,n\right)} \end{array} \end{array}\right] \end{array}.$$

Therefore, by jointly optimizing $\Phi_{xx}$ and $\Phi_{yy}$, the diffraction efficiency and polarization state of each selected diffraction order can be controlled simultaneously.

To demonstrate the design principle, four target diffraction modes are defined. Each mode corresponds to a prescribed combination of diffraction orders and polarization states. Modes 1 and 2 generate linearly polarized outputs at 45° and 135° within the (0, ± 1) diffraction orders, respectively, while Mode 3 produces a pair of orthogonal linear polarization states in the symmetric (± 1, 0) orders; Mode 4 further extends the design to higher‐order diffraction channels (± 2, 0) and (0, ± 2), whose polarization states form an approximately tetrahedral distribution on the Poincaré sphere [32, 33], given by [1, 0.9428, 0, −0.333]^Τ^, [1, −0.4714, 0.8165, −0.333]^Τ^, [1, −0.4714, −0.8165, −0.333]^Τ^ and [1, 0, 0, 1]^Τ^. This tetrahedral arrangement provides a substantial and approximately uniform angular separation between the polarization states on the Poincaré sphere, thereby reducing polarization similarity and crosstalk among the diffraction channels.

For each target mode, the phase distributions $\Phi_{xx}\left(x_{p},y_{p}\right)$ and $\Phi_{yy}\left(x_{p},y_{p}\right)$ are optimized on a discrete 10 × 10 pixel grid. Since each pixel contains two independent phase variables, the super‐pixel has a total of 200 design variables. A particle swarm optimization (PSO) algorithm combined with random perturbations is employed to search this high‐dimensional phase space [34].

To map the optimized phase distribution onto manufacturable structures while maintaining high diffraction efficiency and polarization fidelity, we construct a silicon meta‐atom phase library using single‐crystalline silicon nanopillars on a SiO_2_ substrate. The designed meta‐atoms are silicon rectangular nanopillars with a fixed height of *H* = 900 nm, a lattice period of *P* = 800 nm. Using the finite‐difference time‐domain (FDTD) method, we perform parametric sweeps of the lateral dimensions *L* and *W* over the range of 150–650 nm, extracting the transmission phases $\Phi_{xx}$ and $\Phi_{yy}$, as well as the transmission amplitudes $t_{xx}$ and $t_{yy}$, under *x*‐ and *y*‐polarized illumination, as shown in Figure 3a–e. The resulting library provides nearly 2π phase coverage for both polarization channels while maintaining high transmittance. For each target phase pair $\left(\Phi_{xx}^{\text{opt}},\Phi_{yy}^{\text{opt}}\right)$, the corresponding nanopillar is selected by minimizing the phase mismatch:

(11) $$\begin{array}{c} d=\sqrt{{\left(\Phi_{xx}^{\text{opt}}-\Phi_{xx}\right)}^{2}+{\left(\Phi_{yy}^{\text{opt}}-\Phi_{yy}\right)}^{2}} \end{array}.$$

**FIGURE 3:**
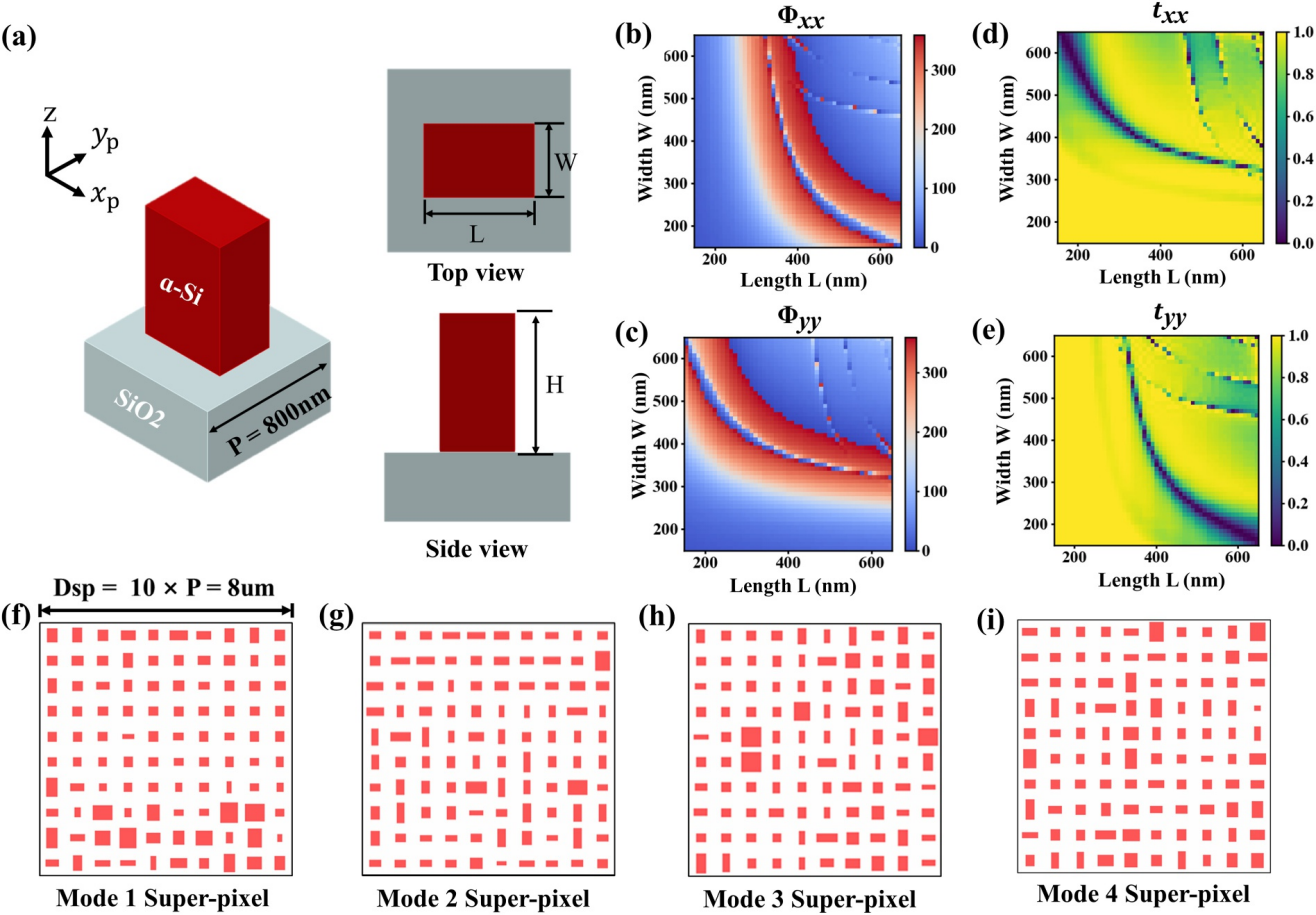
Unit cell of the metasurface and mode super‐pixels. (a) Schematic illustration of the nanopillar. Showing the 3D perspective view, top view, and side view. (b–e) Simulated phase shifts and transmission amplitudes for *x*‐ and *y*‐polarized light as functions of pillar length *L* and width *W*. (f–i) Spatial arrangement of the meta‐atoms for Modes 1–4, respectively. The total size of each super‐pixel is *D*sp = 10 × *P* = 8 μm.

The optimal nanopillar is selected from the phase library using a layered threshold‐screening mechanism based on transmittance, which suppresses the quantization error introduced by discretization while ensuring phase‐matching accuracy.

Finally, the matched meta‐atoms are spatially arranged according to the 10 × 10 phase distributions to form the four super‐pixels, with the corresponding structural dimension maps shown in Figure 3f–i. By imposing multi‐objective constraints on both the phase profile and the polarization state in Fourier space, this method achieves the co‐optimization of diffraction efficiency, polarization fidelity, and channel orthogonality, demonstrating good generality and scalability. Further details of the optimization procedure are provided in Supporting Information S1: Section 1.

#### 2.2.2 Spatially Multiplexed Nested Supercell

To spatially integrate the Fourier‐engineered diffraction modes described above, we construct a nested Fourier metasurface (NFM) by arranging different mode super‐pixels into a spatially multiplexed supercell. The complete nested supercell consists of an *M* × *M* arrangement of mode regions, where *M* denotes the number of mode regions along each direction (with *M* = 2 for the configuration demonstrated in this work). Each mode region corresponds to one independently designed diffraction mode. Within each mode region, the corresponding *D*sp = 8 μm mode super‐pixel is periodically repeated *N* × *N* times. Accordingly, the period of the complete nested supercell is $D_{\text{super}}$ = 2*ND*sp. At a given time, only the selected mode region is optically activated, enabling dynamic switching among the *M* ^2^ predesigned diffraction responses (four modes for *M* = 2).

The finite spatial extent of the activated mode region introduces an additional diffraction envelope. Under the rectangular‐aperture approximation, the one‐dimensional aperture transmission can be characterized by a duty ratio $\eta_{x}$, and its Fourier transform is:

(12) $$\begin{array}{c} F\left\{t\left(x\right)\right\}\left(f_{x}\right)=\int_{-\infty }^{\infty }t\left(x\right)\;e^{-i2\pi f_{x}x}\;dx=\eta_{x}D_{\text{super}}\;\text{sinc}\left(\eta_{x}D_{\text{super}}f_{x}\right) \end{array},$$

where $D_{\text{super}}$ is the period of the rectangular aperture and sinc(*u*) = sin(π*u*)/(π*u*). For the two‐dimensional NFM, the activated mode region can be approximated by rectangular windows along both *x*‐ and *y*‐directions, resulting in a separable sinc‐type envelope in the far field. This envelope modulates the amplitudes of the superlattice diffraction orders and therefore determines how the diffracted power is distributed among the principal peak and its neighboring side orders. A detailed definition of the aperture function, duty ratio, and corresponding side‐order power distribution is provided in Supporting Information S1: Section 2.

The far‐field response of the NFM can be understood through a two‐scale Fourier description. Under the Fraunhofer approximation and weak inter‐super‐pixel coupling, the total far‐field distribution can be approximately expressed as:

(13) $$\begin{array}{c} E_{\text{out}}\left(f_{x},f_{y}\right)={\frac{1}{M}\cdot E}_{\text{sp}}\left(f_{x},f_{y}\right)\;\cdot \;AF_{\text{super}}\left(f_{x},f_{y}\right), \end{array}$$

where the factor 1/*M* accounts for the amplitude dilution of the principal order caused by the reduced effective illuminated area. The element factor $E_{\text{sp}}$ is determined by the locally Fourier‐optimized mode super‐pixel, whereas $AF_{\text{super}}$ is the array factor associated with the spatially nested supercell lattice. The former determines the target diffraction direction and polarization state encoded in each mode, while the latter governs the positions of additional superlattice diffraction orders. Their amplitudes are further weighted by the finite‐aperture sinc envelope described above.

Importantly, the enlarged nested periodicity does not redefine the locally designed diffraction direction. For a mode super‐pixel with target spatial frequency ($f_{x0},f_{y0}$), the corresponding propagation direction is determined by:

(14) $$\begin{array}{c} \sin\;\theta_{x}=\lambda f_{x0},\sin\;\theta_{y}=\lambda f_{y0}. \end{array}$$

After embedding the mode super‐pixel into the nested architecture, additional superlattice orders appear around ($f_{x0},f_{y}$). Instead, it introduces additional superlattice diffraction orders around this target spatial frequency. Therefore, the diffraction peaks of the complete NFM can be approximated as:

(15) $$\begin{array}{c} f_{x}=f_{x0}+\frac{dp}{D_{\text{super}}},f_{y}=f_{y0}+\frac{dq}{D_{\text{super}}},dp,dq\in Z \end{array}.$$

Thus, the nested periodicity determines the positions of the neighboring diffraction orders, while the finite activation window determines their relative amplitudes through the sinc envelope.

Varying *N* preserves the principal diffraction direction but changes the spacing between neighboring superlattice orders:

(16) $$\begin{array}{c} \Delta f=\frac{1}{D_{\text{super}}}=\frac{1}{{2ND}_{\text{sp}}}. \end{array}$$

As *N* increases, Δf decreases, causing the neighboring diffraction orders to become more densely clustered around the target direction. Their system‐level contribution depends on their angular positions relative to the receiver: side orders falling within the target acceptance window can be collected as useful angular signal components, whereas those coupled into undesired ports contribute to inter‐port crosstalk, and uncollected orders appear as effective power leakage.

This analysis reveals the key physical mechanism of the NFM architecture. The local Fourier‐optimized modal super‐pixel determines the primary diffraction direction, whereas the global nested supercell governs the angular distribution, density, and power allocation of the superlattice side peaks. Therefore, the nested architecture provides an additional design degree of freedom for engineering diffraction‐channel distributions without altering the locally encoded metasurface functionality.

## 3 Results

### 3.1 Fourier‐Engineered Multimode Diffraction

To validate the proposed Fourier‐engineered metasurface framework, we first investigate the static multidimensional diffraction behavior across the four designed operating modes. The metasurface is optimized to support multiple independently engineered diffraction channels with distinct diffraction orders and polarization states, thereby enabling multidimensional diffraction‐channel manipulation within a unified Fourier‐optical platform.

Mode 1 produces a far‐field pattern with energy highly concentrated at the target (0, 1) order (Figure 4a), yielding 45° linearly polarized output with a diffraction efficiency of 44.37% and a polarization fidelity of 0.9970 at 1550 nm. The Poincaré sphere representation (Figure 4e) and Stokes parameter comparison (Figure 4i) confirm excellent agreement with the target polarization, demonstrating high‐precision polarization control. Mode 2, corresponding to the reversed (0, −1) channel, delivers 135° linearly polarized light (Figure 4b) with an efficiency of 45.58% and a fidelity of 0.999, indicating outstanding polarization beam‐splitting performance. Mode 3 realizes dual‐channel polarization multiplexing by simultaneously outputting orthogonal linear polarizations at the (−1, 0) and (1, 0) orders (Figure 4c). The total diffraction efficiency reaches 45.52%, with individual channel efficiencies of 21.87% and 23.54%, and corresponding polarization fidelities of 0.9997 and 0.9932. The orthogonal distribution on the Poincaré sphere (Figure 4g) further validates the multi‐channel polarization decoupling capability. Mode 4 extends to four‐channel higher‐order multiplexing at the (± 2, 0) and (0, ± 2) orders (Figure 4d), achieving a total efficiency of 36.61% and an average fidelity of 0.9773. Owing to the 45° linearly polarized input, the energy distribution among channels is governed by their projection onto the input polarization, resulting in a non‐uniform efficiency profile of 6.26%, 13.04%, 10.47%, and 6.84%. Poincaré sphere analysis (Figure 4h) reveals distinct polarization states approaching the predesigned tetrahedral configuration; despite slightly increased crosstalk from the higher channel count, effective polarization discrimination is preserved.

**FIGURE 4:**
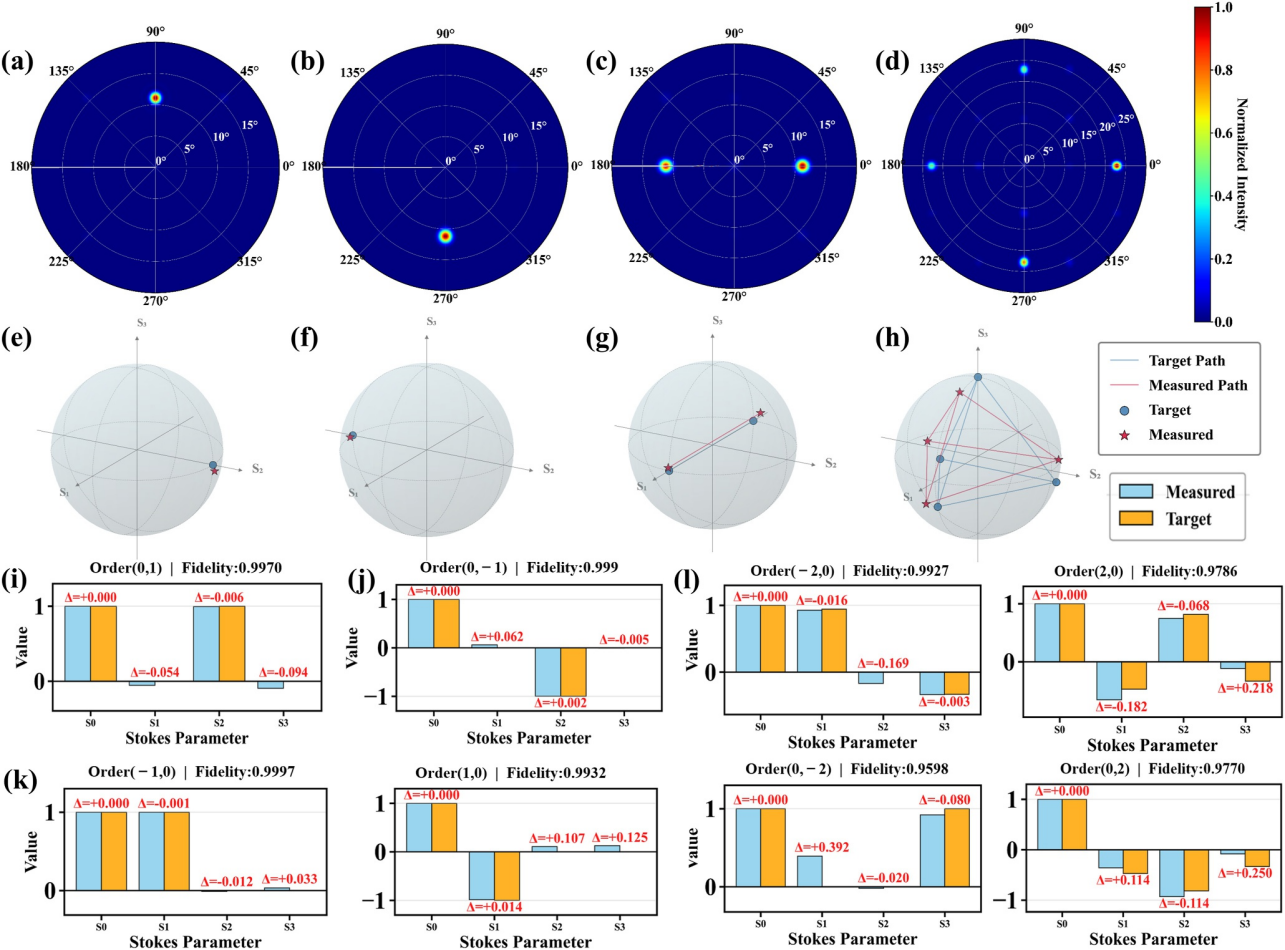
Far‐field pattern, Poincaré sphere and Stokes parameter analysis. (a–d) Simulated far‐field diffraction patterns for Modes 1–4. (e–h) Corresponding polarization states represented on the Poincaré sphere. The blue markers denote target polarization states, while the red markers represent simulated results. (i–l) Comparison of normalized Stokes parameters (S_0_–S_3_) between target and simulated results for each diffraction channel.

Across all four operating modes, the overall device transmittance remains stable at approximately 70%, with corresponding insertion losses of 3.53, 3.41, 3.43, and 4.36 dB. Crosstalk is quantitatively assessed by summing the leakage power into the zeroth‐order and unintended diffraction orders. The resulting crosstalk levels for Modes 1 through 4 are −17.50, −19.03, −16.62, and −14.50 dB, indicating effective channel isolation.

Collectively, these results demonstrate that the Fourier‐coefficient‐based direct optimization approach enables scalable integration from single‐channel to multi‐channel operation within a single metasurface, while maintaining high polarization fidelity and controlled crosstalk. This method offers an effective pathway toward complex polarization multiplexing, multi‐channel information encoding, and dynamic optical systems.

### 3.2 Nested Fourier Metasurface Architecture and Superlattice Diffraction Engineering

Based on the four‐mode routing example (Figure 4), the NFM is constructed as a 2 × 2 array of mode regions, where each region comprises a locally Fourier‐optimized 10 × 10 super‐pixel periodically repeated in an *N* × *N* lattice. This hierarchical architecture enables decoupled control over the far‐field physics: the local super‐pixel fundamentally determines the primary routing direction, while the global supercell parameter *N* governs the angular density of neighboring diffraction sidebands. As predicted by our theoretical model (Section 2.2.2) and validated by increasing *N* from 1 to 8 (Figure 5), a larger *N* significantly reduces the angular spacing between adjacent superlattice orders. Crucially, rather than generating new principal orders, this process compactly clusters satellite side peaks around the intrinsic target spatial frequency defined by the local phase profile. Consequently, for the *N* = 8 configuration, multiple side peaks effectively fall within the receiver limited acceptance window (± 1.1°), which systematically reshapes the angular‐channel distribution and significantly enhances useful power collection through multi‐angle reception, successfully demonstrating momentum‐space multidimensional routing.

**FIGURE 5:**
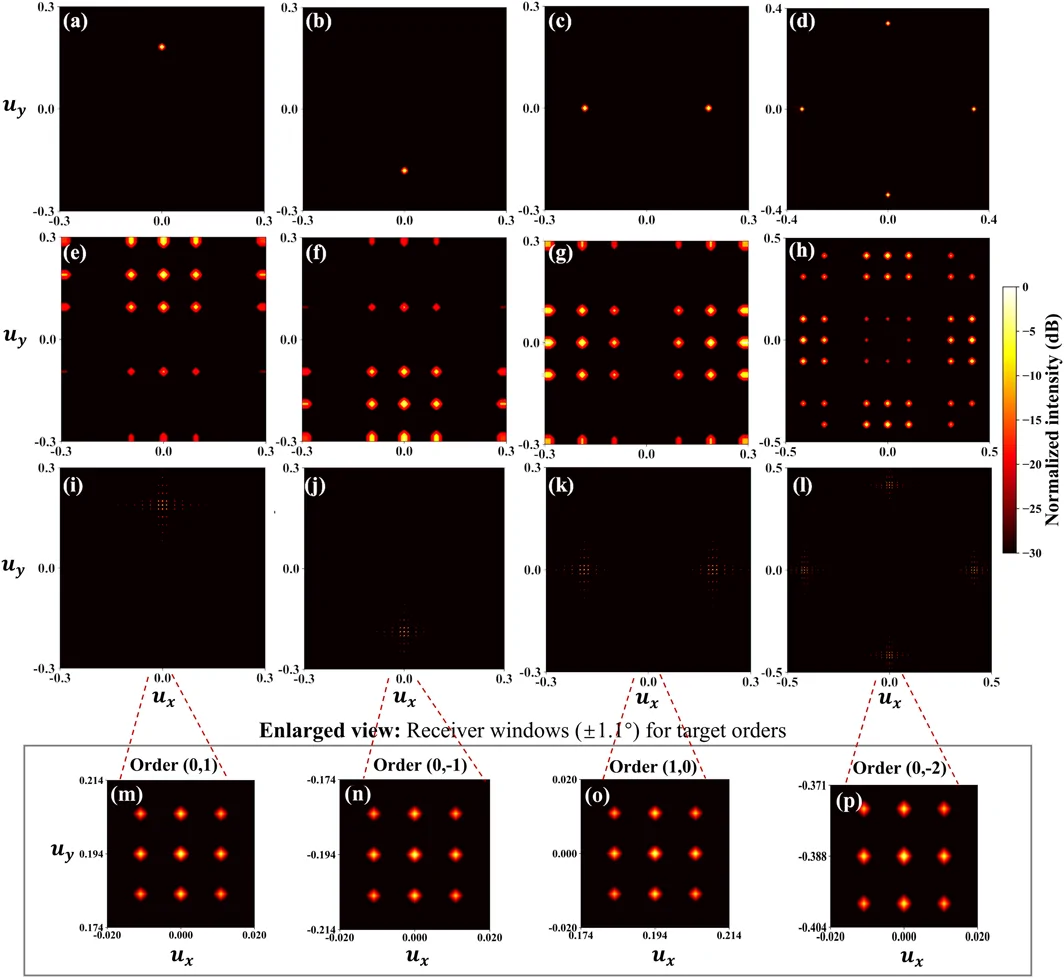
Superlattice diffraction behavior of the nested Fourier metasurface under different *N* configurations. Panels (a–d) show the reference far‐field diffraction patterns of modes 1–4, respectively. Panels (e–h) present the far‐field distributions of the nested four‐mode metasurface with *N* = 1 under excitation of modes 1–4, respectively, while panels (i–l) correspond to the *N* = 8 configuration. Compared with the *N* = 1 configuration, the *N* = 8 configuration produces denser spatial‐frequency sampling and clustered side peaks around the target diffraction directions, thereby enhancing the useful power collection within the angular reception window. The color scale represents the normalized far‐field intensity (dB). Panels (m–p) provide enlarged views of the receiver windows (± 1.1°) for the target orders (0, 1), (0, −1), (1, 0), and (0, −2), illustrating the clustered side peaks produced by the *N* = 8 configuration.

To rigorously validate these momentum‐space predictions and evaluate the true device performance, we performed full‐wave electromagnetic simulations using the finite‐difference time‐domain (FDTD) method, incorporating a finite‐sized NFM metasurface array to capture realistic electromagnetic interactions, including inter‐unit‐cell coupling and local field effects.

As detailed in Table 1, under individual mode activation at 1550 nm, the simulated diffraction efficiencies within the ± 1.1° receiver window are 36.53%, 37.13%, 36.49%, and 29.34% for Modes 1–4, respectively, with corresponding crosstalk levels of −18.15, −18.98, −16.18, and −14.50 dB. By effectively aggregating the clustered sidebands, this architecture yields a useful power gain of approximately 5 dB compared to collecting only the main diffraction order. Furthermore, the residual leakage to higher‐order sidebands remains below −6 dB relative to the useful power, and the simulated sideband distributions are in excellent agreement with the theoretical sinc‐envelope model established in Section 2.2.2. This full‐wave validation strongly corroborates the physical soundness and quantitative predictability of the nested superlattice design.

**TABLE 1:** Performance comparison of the four operation modes in the OA‐NFM platform.

Parameter	Mode 1 (1‐to‐1)	Mode 2 (1‐to‐1)	Mode 3 (1‐to‐2)	Mode 4 (1‐to‐4)
Target diffraction order (s)	(0, +1)	(0, −1)	(−1, 0), (+1, 0)	(± 2, 0), (0, ± 2)
Target routing direction	11.2°	11.2°	11.2°	22.8°
Isolated mode efficiency	44.37%	45.58%	45.42%	36.61%
Receiver efficiency^a^	36.53%	37.13%	36.49%	29.34%
Main order efficiency	10.54%	10.69%	10.49%	8.47%
On‐axis sidebands power (dB)^b^	−3.85 dB	−3.84 dB	−3.87 dB	−3.85 dB
Diagonal sidebands power (dB)	−7.70 dB	−7.69 dB	−7.61 dB	−7.70 dB
Efficiency retention (window/isolated)	82.3%	81.5%	80.3%	80.1%
Useful power gain (dB)^c^	5.22	5.23	5.14	4.99
Residual leakage ratio (dB)	−6.68	−6.43	−6.11	−6.06
Max delay spread (ps)	0.84	0.84	0.84	2.0

^a^ Total useful efficiency collected within the ± 1.1° receiver acceptance window, calculated via full‐wave FDTD simulations.

^b^ Power relative to the main diffraction order. The theoretical values predicted in Section 2.2.2 are approximately −3.92 dB for on‐axis orders (∣*dp*∣ = 1, *dq* = 0 or *dp* = 0, ∣*dq*∣ = 1) and −7.84 dB for diagonal sidebands orders (∣*dp*∣ = ∣*dq*∣ = 1).

^c^ Power gain achieved by multi‐angle reception of clustered sidebands within the window compared to collecting only the main order.

While the aggregation of multidimensional angular channels maximizes power transfer, it inevitably introduces multipath delay spread. However, assuming a representative short‐reach free‐space transmission distance of 5 cm, the maximum temporal delays are merely 0.84 ps for Modes 1–3 and 2.0 ps for Mode 4. These values are an order of magnitude smaller than the 20 ps bit period of a standard 50 Gbps NRZ‐OOK optical link. Consequently, the inter‐symbol interference (ISI) penalty arising from temporal dispersion is entirely negligible for high‐speed short‐reach applications, such as chip‐to‐chip communications, board‐to‐board optical interconnects, and short‐distance free‐space optical switching in data centers.

Furthermore, this spatially multiplexed layout integrates multiple independently optimized functional regions on a single metasurface, selectively activated by the DMD to enable on‐demand switching among predefined routing states. Several other metasurface platforms also adopt DMD‐enabled optical addressing. Recent optical‐addressing metasurface spatial‐light modulators (OA‐MSLMs) pursue pixel‐level or subwavelength‐scale programmability and therefore provide substantially higher spatial reconfiguration freedom [22]. In contrast, the present OA‐NFM adopts function‐level addressing of pre‐engineered mode regions, trading pixel‐level programmability for relaxed addressing resolution and direct access to optimized diffraction–polarization functions. Given these design features, it targets optical‐communication routing scenarios prioritizing high diffraction efficiency and low crosstalk. Under this paradigm, routing functions are mapped onto macroscopic mode‐regions optically selected by the DMD, maintaining favorable collection efficiency and inter‐mode isolation and offering a scalable, practical pathway toward programmable optical interconnects.

### 3.3 Communication System Demonstration

To demonstrate the feasibility of the proposed metasurface architecture for free‐space optical communication applications, a basic NRZ‐OOK modulation scheme is evaluated in system‐level simulations under different high‐speed data rates. Communication‐link parameters, including channel gain, insertion loss, and inter‐channel crosstalk, are extracted from full‐wave FDTD simulations based on the optical power collected within the designated receiver windows. The reported insertion loss and BER characterize the intrinsic OA‐NFM channel. External losses associated with DMD reflectivity/diffraction efficiency, 4f‐system transmission, finite addressing contrast, aperture clipping, and alignment errors are not included in the present model. These terms will contribute to the end‐to‐end experimental link budget and will be considered in future experimental implementation.

At the transmitter, a pseudorandom bit sequence (PRBS) is converted by an arbitrary waveform generator (AWG) into an electrical driving signal, which drives a Mach–Zehnder modulator (MZM) to modulate the optical carrier from a continuous‐wave (CW) laser, generating a high‐speed NRZ‐OOK optical signal. The modulated signal is then directed onto the DMD, where different routing patterns are loaded to selectively configure the spatial routing state, enabling unicast, dual‐channel, or four‐channel transmission through the corresponding quasi‐static channels. At the receiver, a multi‐pixel photodetector array (PD array) is placed at the far‐field output plane, with each detection element angularly aligned to one target output channel. A rectangular aperture is placed directly in front of the detector, with its dimensions determined by the required ± 1.1° acceptance angle in both horizontal and vertical directions, following the geometric relation D≈2Ltanθ, where D is the aperture dimension, L is the propagation distance from the metasurface to the detector, and θ = 1.1°. In the simulation, the diffraction efficiency is obtained by integrating the far‐field power within a ± 1.1° window centered on the corresponding target diffraction peak. In this model, the detector, under the restriction of the rectangular aperture, collects all power within the window, including the target diffraction peak and adjacent superlattice sidebands, which are jointly collected and detected by the same photodetector. The received signal is then directly detected, sampled, and subjected to threshold decision, with the overall system performance evaluated using eye diagrams and bit‐error‐rate (BER) analysis. It is worth noting that the DMD operates on a microsecond timescale, serving solely to configure and latch the routing state prior to data transmission, while bit‐level high‐speed NRZ‐OOK modulation is entirely handled by the independent MZM. The two are fully decoupled in both timescale and function.

Figure 6 presents the BER performance of all four operating modes at 1500 nm, 1550 nm, and 1600 nm. In the simulations, no equalization is applied at the receiver, including no 5‐tap FFE, with signal recovery based solely on hard‐decision threshold detection, so as to directly assess the impact of the modeled insertion loss, crosstalk, and bandwidth limitations on system performance. Under these conditions, all four modes maintain BER below the HD‐FEC threshold (10^−3^) at data rates up to 25 Gbps across the entire 1500–1600 nm band, with open eye diagrams confirming good signal integrity. At 1550 nm, each channel sustains BER at or below the FEC threshold as the data rate is increased to 40 Gbps, demonstrating the system’s robust high‐speed transmission capability. Single‐channel modes (Modes 1 and 2) exhibit superior BER performance compared to multi‐channel modes (Modes 3 and 4). In the multi‐channel case, energy distribution and inter‐channel coupling introduce modest performance degradation. Nevertheless, all modes remain within acceptable limits, and the performance variation among them is primarily governed by the diffraction efficiency and crosstalk level of each channel. Collectively, these results confirm that the proposed architecture enables stable, low‐crosstalk, multi‐channel optical routing across the full 1500–1600 nm telecom band.

**FIGURE 6:**
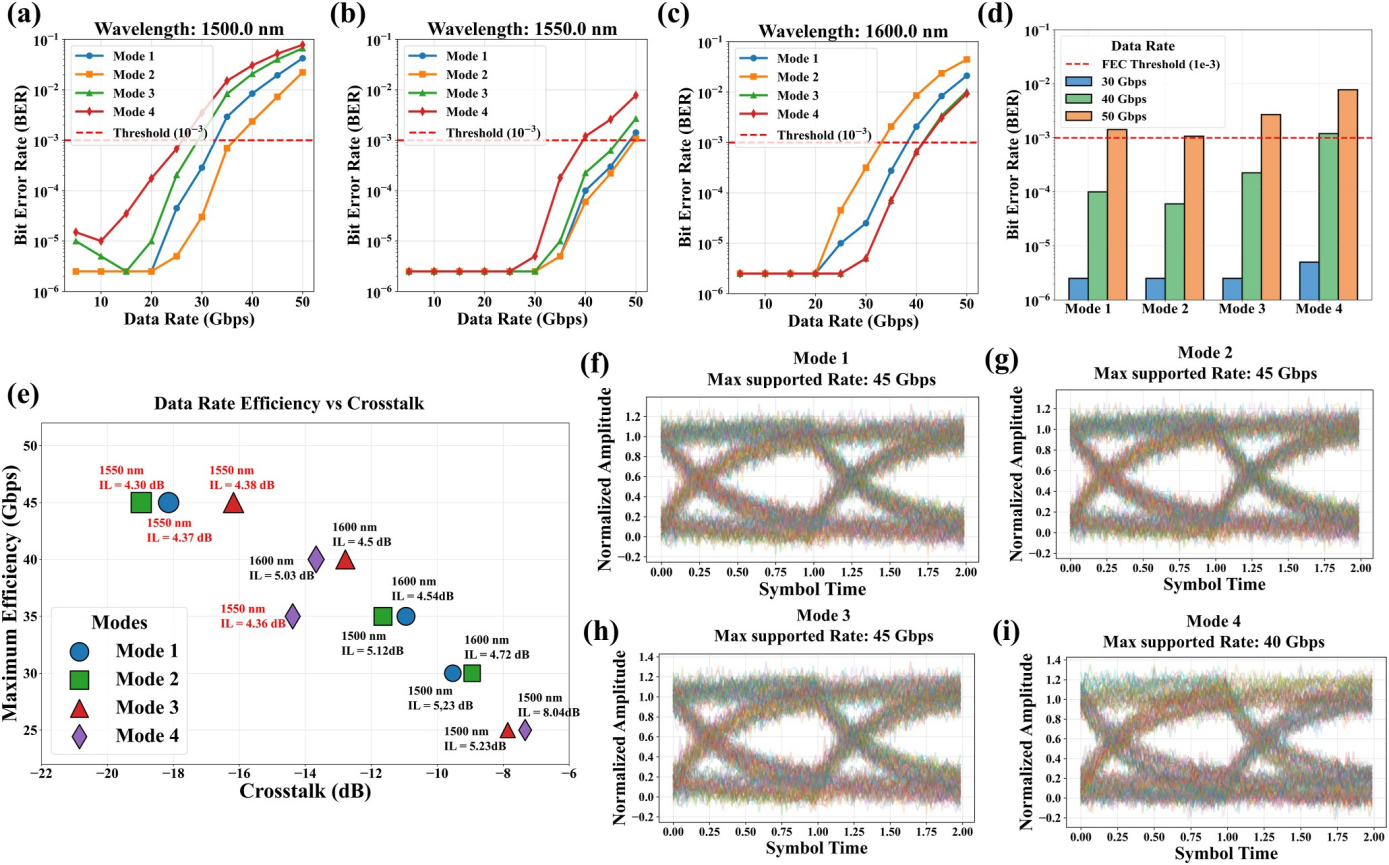
System‐level performance of the four diffraction modes under NRZ modulation at different wavelengths. (a–c) BER as a function of data rate for Mode 1–Mode 4 at wavelengths of 1500, 1550, and 1600 nm, respectively. (d) BER comparison of different modes at data rates of 30, 40, and 50 Gbps. (e) Relationship between the maximum supported data rate and crosstalk for the four modes at different wavelengths, with insertion loss (IL) labeled beside each data point. (f–i) Normalized eye diagrams of Modes 1–4 at 1550 nm. The maximum supported rates are 45 Gbps for Modes 1–3 and 40 Gbps for Mode 4.

## 4 Discussion

The proposed OA‐NFM platform enables programmable multidimensional diffraction‐channel engineering within a compact metasurface architecture. Through Fourier‐coefficient optimization, multiple diffraction orders and polarization states are simultaneously controlled within a unified design framework, extending metasurface functionality beyond conventional single‐channel beam steering. The wiring‐free optical‐addressing mechanism eliminates dense electrical interconnects, while the nested Fourier metasurface strategy introduces hierarchical superlattice diffraction engineering for controllable angular‐channel clustering. As a proof‐of‐concept system demonstration, an NRZ‐OOK free‐space optical communication model confirms stable multichannel routing with acceptable BER performance under metasurface‐level insertion‐loss and crosstalk conditions, establishing the physical connection between diffraction‐sideband distributions, angular channel spacing, and communication‐channel degradation.

The NFM architecture further provides two complementary routes toward scalability. At the local level, the accessible momentum‐space channels are determined by the Fourier‐engineered mode super‐pixel. For a square super‐pixel with period $D_{\text{sp}}=N_{p}\times P$, the propagating diffraction orders satisfy:

(17) $$\begin{array}{c} m^{2}+n^{2}\leq {\left(\frac{D_{\text{sp}}}{\lambda }\right)}^{2}. \end{array}$$

For the design parameters used in this work ($N_{p}$ = 10, *D*sp = 8 μm, *λ* = 1550 nm), this yields $m^{2}+n^{2}\leq 26.63$, giving 89 propagating two‐dimensional diffraction‐order pairs (*m*, *n*), including the zero order. These orders define an accessible momentum‐space basis from which multiple diffraction channels can be selected and optimized. Increasing $N_{p}$ expands this basis and provides additional degrees of freedom for redistributing optical power among target channels.

At the global level, expanding the mode‐region arrangement from the demonstrated 2 × 2 layout to a general *M* × *M* configuration accommodates up to *M* ^2^ independently addressable spatial modes. As *M* increases, the superlattice angular spacing Δθ decreases, allowing more adjacent superlattice orders to fall within a fixed receiver acceptance window *Ω* (where *Ω* denotes the set of all superlattice diffraction orders (dp,dq) within the ± 1.1° window). The window‐collection efficiency is given by:

(18) $$\begin{array}{c} \eta_{\text{window}}\left(M\right)=\frac{\eta_{\text{iso}}}{M^{2}}\sum_{\left(dp,dq\right)\in \Omega }{\text{sinc}}^{2}\left(\frac{dp}{M}\right){\text{sinc}}^{2}\left(\frac{dq}{M}\right), \end{array}$$

where $\eta_{\text{iso}}$ denotes the isolated mode efficiency (for Mode 1, $\eta_{\text{iso}}$ = 44.37%) and $\eta_{\text{main}}\left(M\right)={\eta_{\text{iso}}/M}^{2}$ is the principal‐order efficiency. This expression accounts for two competing effects: the principal‐order power dilution (1/*M* ^2^) and the growing number of sidebands collected inside the reception window. The useful power gain relative to collecting only the principal order is defined as:

(19) $$\begin{array}{ll} G_{\text{win}}\left(M\right) & =10\;\log_{10}\left[\frac{\eta_{\text{window}}\left(M\right)}{\eta_{\text{main}}\left(M\right)}\right]\\ & =10\;\log_{10}\left[\sum_{\left(dp,dq\right)\in \Omega }{\text{sinc}}^{2}\left(\frac{dp}{M}\right){\text{sinc}}^{2}\left(\frac{dq}{M}\right)\right]. \end{array}$$

Numerically $G_{\text{win}}\left(M\right)$ increases from 5.16, 8.66, 11.15, and 13.09 dB for the 2 × 2, 3 × 3, 4 × 4, and 5 × 5 configurations, respectively (Supporting Information S1: Table S1). Across all configurations, the window‐collection efficiency $\eta_{\text{window}}\left(M\right)$ retains more than 80% of $\eta_{\text{iso}}$, confirming that the receiver window captures the dominant fraction of the diffracted power regardless of array size. Full‐wave FDTD simulations validate these predictions. As shown in Figure 7, the far‐field patterns of the activated Mode 1 region under 2 × 2, 3 × 3, and 5 × 5 arrangements show progressively denser superlattice sidebands around the (0, 1) target order, with simulated window‐collection efficiencies of 36.53%, 36.05%, and 36.14%, respectively, in close agreement with the theoretical values. Although the principal‐order efficiency decreases as $1/M^{2}$, more diffraction orders fall within the reception window and enable optical‐power redistribution, so that the $\eta_{\text{window}}\left(M\right)$ of the NFM structure remains nearly constant across different configurations.

**FIGURE 7:**
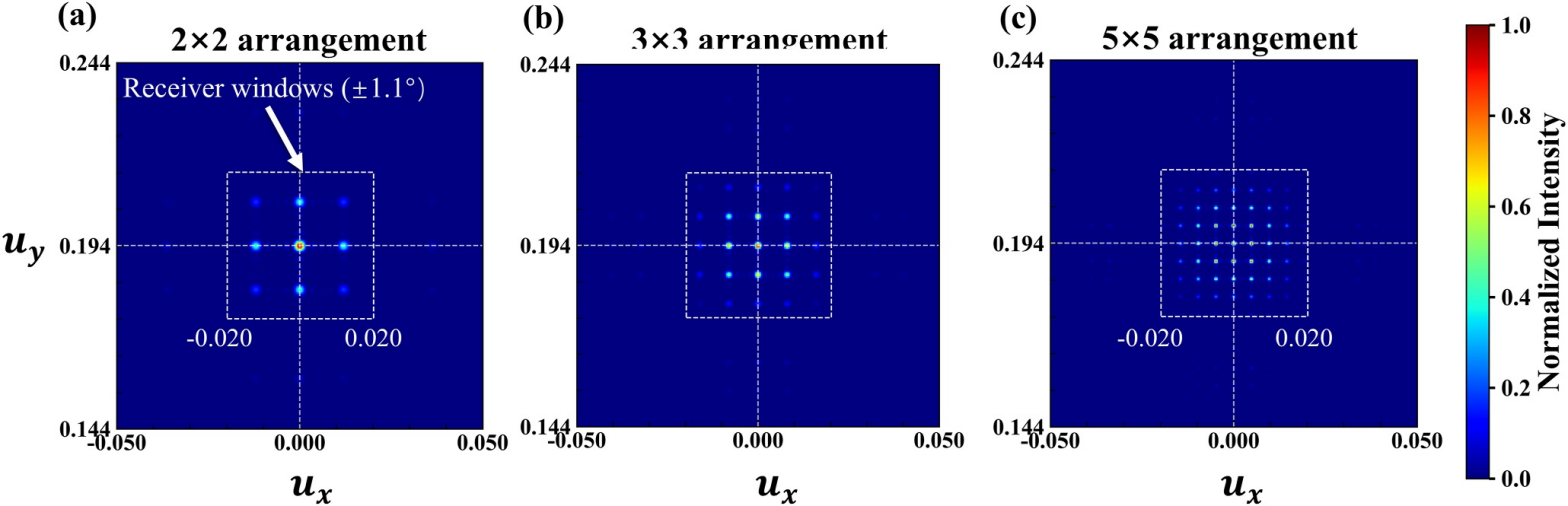
Far‐field diffraction patterns of the activated Mode 1 region under different mode‐region arrangements, showing the target (0,  1) order. Panels (a–c) correspond to *M* × *M* arrangements of 2 × 2, 3 × 3, and 5 × 5, respectively. The zoomed‐in views highlight the angular distribution of the principal peak and its neighboring superlattice sidebands within the receiver window.

The NFM architecture offers two potential scaling routes: increasing diffraction‐order degrees of freedom within mode super‐pixels and expanding the spatial array to larger *M* × *M* configurations. Their combination could enable higher‐dimensional diffractive routing, subject to constraints from mode count, power budget, crosstalk, receiver resolution, and optical addressing. As summarized in Table 2, different switching technologies exhibit distinct trade‐offs in reconfiguration speed, routing scale, insertion loss, crosstalk, and implementation complexity. The present OA‐NFM demonstrates four functional routing modes in a 2 × 2 arrangement, while the larger‐array analysis, using Mode 1 as a representative case, demonstrates the predicted superlattice‐diffraction scaling behavior up to a 5 × 5 arrangement. This analysis provides evidence for the spatial scalability of the NFM architecture (see Supporting Information S1: Section 3 for details), and the same design strategy can in principle be applied to larger‐array configurations of the other target diffraction modes (e.g., Modes 2–4), while larger *M* × *M* multimode configurations remain a potential extension of the present framework.

**TABLE 2:** Comparison of representative programmable optical routing and switching platforms.

	3D MEMS [35, 36]	LCoS‐WSS [37, 38, 39, 40]	EO‐MZI [41, 42, 43]	AWGR [3, 44, 45, 46]	SOA [3, 47, 48, 49, 50]	OA‐NFM
Operating principle	Mechanical beam steering	LC phase modulation	EO interference	Wavelength routing	Optical gain gating	Fourier diffraction + polarization multiplexing
Reported routing scale	100 × 100–1100 × 1100	1 × N	16 × 16–32 × 32	64 × 64–512 × 512	8 × 8–32 × 32	4 functional modes (2 × 2); Mode‐1 scaling studied to 5 × 5
Wiring complexity	2592 mirrors and 10,368 control channels [36]	Pixel‐array electrical addressing	144 MZIs and 288 electrode pads (32 × 32) [43]	Passive wavelength routing; no electrical interconnect	192 SOA drive connections for 16 × 16 [49]	Optical addressing; no electrical interconnect
Dynamic reconfiguration speed	1–10 ms	10–100 ms	1–10 ns	∼1 ns	10 ps–1 ns	∼10–100 μs
Insertion loss (dB)	1–4	3–6	5–20	2–8 dB component‐level; 5–20 dB system‐level	Gain‐compensated loss: 1–3	∼3–5 (intrinsic metasurface IL)
Crosstalk (dB)	< −50	−25 to −35	−10 to −20	−15 to −25	−25 to −40	−14 to −19

## 5 Conclusion

In this work, we proposed and numerically demonstrated an optically addressed nested Fourier metasurface (OA‐NFM) platform for programmable multidimensional optical routing and diffraction‐channel manipulation. By combining Fourier‐coefficient‐based optimized design with dynamic DLP optical addressing, the proposed architecture enables reconfigurable control of diffraction channels and polarization states without dense subwavelength electrical interconnects.

The key innovation is the nested Fourier metasurface strategy, in which diffraction functionalities are encoded into mode regions, relaxing the optical‐resolution requirement while enabling hierarchical superlattice diffraction engineering. The locally optimized Fourier modes achieve high diffraction efficiencies (∼45% for single‐channel modes) with polarization fidelities exceeding 0.99 at 1550 nm. For the designed 2 × 2 nested configuration, aggregation of the first‐order superlattice sidebands yields window‐collection efficiencies up to approximately 37% (36%–37% for Modes 1–3), confirming effective recovery of the diffracted power redistributed by the superlattice. In the communication‐level validation, all routing modes maintain BER below the hard‐decision FEC threshold (10^−3^) at 25 Gbps across the investigated 1500–1600 nm wavelength range, with clearly open eye diagrams at 40 Gbps at 1550 nm. These simulations consider only the metasurface response and exclude losses and nonidealities associated with the DMD and projection optics. Moreover, the *M* × *M* scalability analysis shows that the window‐collection efficiency remains above 80% of the isolated‐mode efficiency as the number of addressable spatial modes increases.

Overall, this work establishes a unified framework that integrates Fourier‐engineered metasurface physics, optical addressing, superlattice diffraction engineering, and multidimensional optical information processing. The demonstrated scaling behavior suggests that the framework could be further explored for larger and more complex routing architectures. Beyond optical switching and free‐space optical interconnects, the proposed approach may provide a basis for future field‐programmable nanophotonic systems capable of dynamically manipulating multiple optical degrees of freedom.

## Author Contributions

**Jing Guo:** conceptualization, methodology, software, data curation, investigation, validation, writing – original draft, formal analysis, visualization. **Qinglei Zhao:** conceptualization, methodology, software, data curation, investigation, validation, funding acquisition, project administration, resources, supervision. **Jingrui Yang:** conceptualization, methodology, software, data curation, investigation, validation, formal analysis. **Zongyi Liu:** conceptualization, methodology, software, data curation, investigation, validation, formal analysis. **Mingdong Zhu:** conceptualization, methodology, data curation, software, supervision, investigation. **Shuai Liu:** conceptualization, methodology, software, data curation, investigation. **Chao Li:** conceptualization, methodology, software, data curation, investigation, validation, supervision. **Shuxin Wang:** supervision, data curation, software, methodology, conceptualization, investigation. **Qinglong Hu:** conceptualization, methodology, software, data curation, supervision. **Fengwei Guan:** conceptualization, methodology, software, data curation, supervision. **Qiang Liu:** conceptualization, methodology, validation, investigation, software, formal analysis, supervision, data curation, writing – review and editing, funding acquisition, project administration, resources. **Qi Song:** conceptualization, investigation, methodology, validation, software, formal analysis, data curation, supervision, writing – review and editing, writing – original draft, funding acquisition, project administration, resources.

## Funding

This study was supported by the Hunan Provincial Department of Agriculture and Rural Affairs Project, Grants XIANG CAI JIAN ZHI (2025) No. 137 and XIANG CAI JIAN ZHI (2026) No. 45; Hunan Agricultural Science and Technology Innovation Fund Project, Grants 2025CX35 and 2026CX05.

## Conflicts of Interest

The authors have nothing to report.

## Supporting information


Supporting Information S1

## Data Availability

The data that supports the findings of this study are available in the supplementary material of this article.
