# Supplementary material for: Programmable Multidimensional Diffraction Engineering via an Optically Addressed Nested Fourier Metasurface
Source: Nanophotonics. 2026 Oct 7;15(19):e70309. doi: 10.1002/nap2.70309 (PMC13645062; doi:10.1002/nap2.70309)
Supplement: Supplementary file 1 [file NAP2-15-e70309-s001.docx]

**Supporting Information for**

**Programmable Multidimensional Diffraction Engineering via an Optically Addressed Nested Fourier Metasurface**

Jing Guo ^1,2^, Qinglei Zhao ^1^, Jingrui Yang ^3^, Mingdong Zhu ^3,4^, Zongyi Liu ^1,2^, Shuai Liu ^1,2^, Chao Li ^5^, Shuxin Wang ^1^, Qinglong Hu ^1^, Fengwei Guan ^1^, Qiang Liu ^1,^* and Qi Song ^6,^*

1	Changchun Institute of Optics, Fine Mechanics and Physics, Chinese Academy of Sciences, Changchun 130033, China;

2	University of Chinese Academy of Sciences, Beijing 100039, China

3	Yuelushan Laboratory, Changsha 410128, China

4	State Key Laboratory of Hybrid Rice, Hunan Academy of Agricultural Sciences, Changsha 410125, China;

5	Qualcomm Inc., San Diego, CA 92121, USA; snappylichao@gmail.com

6	Soleilware Photonics LLC., San Diego, CA 92131, USA

*	Correspondence: liuqiang@ciomp.ac.cn (Q.L.); songq@sibet.ac.cn (Q.S.)

**Section 1: Design and optimization procedure of Fourier engineered metasurface with multidimensional channels**

1. **Library sweeping:** The meta-atom library was constructed at the design wavelength of 1550 nm via parameter sweeps of rectangular nanostructures. During the optimization, the lattice period was fixed at 800 nm and the nanostructure height was set to 900 nm. The lengths and widths of the rectangular meta-atoms were independently scanned from 150 nm to 650 nm with a step size of 10 nm. For each geometry, the complex transmission responses, including $t_{xx}$, $t_{yy}$, $\text{Φ}_{\text{x}\text{x}}$ and $\text{Φ}_{\text{yy}}$, were recorded to establish the polarization-dependent phase library. Additional uniformity constraints were introduced for modes containing multiple target diffraction orders in order to improve the power balance among different diffraction channels. **Figure S1** shows the width and length with respect to output phase.
2. **Fourier phase design:** generate initial phase distribution and optimize to obtain $\text{Φ}_{\text{x}\text{x}}^{\text{opt}}\text{(}\text{x}_{\text{p}}\text{,}\text{ }\text{y}_{\text{p}}\text{)}$ and $\text{Φ}_{\text{y}\text{y}}^{\text{opt}}\text{(}\text{x}_{\text{p}}\text{,}{\text{ }\text{y}}_{\text{p}}\text{)}$.
3. **Polarization/Phase matching and optimization:** Polarization and Phase matching are utilized to initialize Fourier metasurface design. To further improve the phase-matching accuracy, the updated phase library was linearly interpolated to achieve an effective parameter interval of 5 nm while preserving the same phase-matching mechanism. Meta‑atoms that satisfy a phase error smaller than 0.06 rad and transmission efficiency *T* > 0.8 are preferentially selected.

The optimization proceeds according to the following iterative framework:

***1. Initialization.*** *Random phase distributions*$\text{Φ}_{\text{xx}}^{\text{(0)}}\text{(}\text{x}_{\text{p}}\text{,}\text{y}_{\text{p}}\text{)}$ *and* $\text{Φ}_{\text{yy}}^{\text{(0)}}\text{(}\text{x}_{\text{p}}\text{,}\text{y}_{\text{p}}\text{)}$ *are generated.*

***2. Fourier Coefficient Calculation.*** *The diffraction coefficients* $\text{c}_{\text{xx}}^{\text{(m, n)}}$ *and* $\text{c}_{\text{y}\text{y}}^{\text{(m, n)}}$*are computed using* *the Fast Fourier Transform (FFT).*

***3: Cost-Function Evaluation.*** *The optimization minimizes both polarization error and diffraction efficiency deviation:*

$$\begin{aligned} \epsilon=\sum_{i} w_{i}\left. |S_{i}-S_{i}^{\text{target}} \right.|^{2}+\sum_{i} \eta_{i}\left( \eta_{i}^{\text{target}}-\eta_{i} \right)^{2}\#\left( S1 \right) \end{aligned}$$

*where* $S_{i}$ *is the simulated Stokes vector,* $\eta_{i}$*is the diffraction efficiency, and* $w_{i}$ *is a weighting factor.*

***4. Iterative Optimization.*** *Particle swarm optimization (PSO) combined with random perturbation is adopted to seek optimized solutions.*

***5. Termination.*** *Optimization terminates when* $\epsilon<{10}^{-4}$ *or iteration count reaches 300.*

The optimized phase distributions $\text{Φ}_{\text{x}\text{x}}^{\text{opt}}\text{(}\text{x}_{\text{p}}\text{,}\text{y}_{\text{p}}\text{)}$ and $\text{Φ}_{\text{y}\text{y}}^{\text{opt}}\text{(}\text{x}_{\text{p}}\text{,}\text{y}_{\text{p}}\text{)}$ are obtained, as shown in **Figure S2**. This approach enables the generation of the target diffraction orders with a trade-off between diffraction efficiency and orthogonal polarization state fidelity.

**Figure S1.** Simulated phase shifts ($\text{Φ}_{\text{x}\text{x}}$, $\text{Φ}_{\text{y}\text{y}}$) and transmissions ($t_{xx}$, $t_{yy}$) of nanofin blocks as functions of length width.

**Figure S2.** Optimized phase distributions ($\text{Φ}_{\text{x}\text{x}}^{\text{opt}}$, $\text{Φ}_{\text{yy}}^{\text{opt}}$) for modes 1-4.

**Section 2: Design Schemes of the Nested Fourier Metasurface (NFM)**

In this section, we present the structural design and operating principle of the nested Fourier metasurface (NFM), and comparatively analyze the far-field diffraction characteristics of the *N*=1 and *N*=8 configurations, corresponding to different repetition numbers of mode super-pixels within each mode region. The objective is to verify that the proposed 8×8 nested architecture can achieve the intended performance in terms of energy concentration, side-peak engineering, and multi-channel angular multiplexing.

Under the Fraunhofer far-field approximation, the angular spectrum distribution of a metasurface can be described by the spatial Fourier transform of its complex transmission function. Therefore, the far-field diffraction characteristics generated by the nested supercell structure can be analytically understood through its spatial aperture function. Considering a one-dimensional rectangular aperture with width $d_{x}$ within a period $D_{\text{super}}$, the duty ratio is defined as $\eta_{x}=d_{x}/D_{\text{super}}\in\left( 0,1 \right]$, and the corresponding aperture transmission function can be expressed as:

$$\begin{aligned} t\left( x \right)=\text{rect}\left( \frac{x}{\eta_{x}D_{\text{super}}} \right),\#\left( S2 \right) \end{aligned}$$

where $\text{rect}\left( u \right)$ denotes the standard rectangular function, defined as:

$$\begin{aligned} \text{rect}\left( u \right)=\left\{ \begin{aligned} 1，\left| u \right|<\frac{1}{2} \\ 0，\left| u \right|<\frac{1}{2} \end{aligned} \right..\#\left( S3 \right) \end{aligned}$$

Taking the Fourier transform of the aperture function gives the far-field amplitude distribution as:

$$\begin{aligned} \mathcal{\mathcal{F}}\{t\left( x \right)\}\left( f_{x} \right)=\int_{-\infty}^{\infty} t\left( x \right) e^{-i2\pi f_{x}x} dx=\eta_{x}D_{\text{super}}\text{sinc}\left( \eta_{x}D_{\text{super}}f_{x} \right),\#\left( S4 \right) \end{aligned}$$

where the normalized sinc function is defined as:

$$\begin{aligned} \text{sinc}\left( u \right)= \frac{sin\left( \pi u \right)}{\pi u}\text{, }\text{sinc}\left( 0 \right)=1.\#\left( S5 \right) \end{aligned}$$

The activated region of the supercell can be represented by rectangular window functions along both the x- and y-directions. Therefore, the two-dimensional far-field envelope can be factorized into the product of two one-dimensional sinc functions. For a discrete diffraction order shifted by $\left( dp,dq \right)$ with respect to the target order, the corresponding spatial-frequency offsets are determined by the supercell period $D_{super}$. Specifically, the frequency offset along the xxx-direction can be written as $f_{x}=dp/D_{\mathrm{super}}$. The corresponding gating coefficient is defined as:

$$\begin{aligned} C\left( dp,dq \right)=\text{sinc}\left( dp\cdot\eta_{x} \right) \text{sinc}\left( dq\cdot\eta_{y} \right).\#\left( S6 \right) \end{aligned}$$

By normalizing the complex amplitude of the $dp,dq)$ diffraction order to that of the target diffraction component, the corresponding relative power can be expressed as:

$$\begin{aligned} P_{\text{gate}}\left( dp,dq \right)=\left| C\left( dp,dq \right) \right|^{2}=\text{sin}\text{c}^{\text{2}}\left( dp\cdot\eta_{x} \right) \text{sin}\text{c}^{\text{2}}\left( dq\cdot\eta_{y} \right).\#\left( S7 \right) \end{aligned}$$

The above expression indicates that local mode activation within the supercell is equivalent to applying a finite spatial window to an ideal periodic grating. As a result, the target diffraction peak is broadened in the spatial-frequency domain, forming side-peak structures modulated by the sinc envelope. In the proposed design, the nested supercell is arranged as a 2×2 array, and only one mode region is illuminated and activated by the DMD projection at a given time. Therefore, $\eta_{x}=\eta_{y}=0.5$. For the first-order side peaks ($dp$= ±1, $dq$= 0) and ($dp$= 0, $dq$= ±1), the normalized power can be approximated as:

$$\begin{aligned} P_{\text{gate}}\left( \pm1,0 \right)=P_{\text{gate}}\left( 0,\pm1 \right)=\text{sin}\text{c}^{\text{2}}\left( 0.5 \right)=\left( \frac{2}{\pi} \right)^{2}\approx0.4053 \hat{=} -3.92 \text{dB}\text{.}\#\left( S8 \right) \end{aligned}$$

Similarly, the normalized power of the diagonal second-order side peaks can be obtained as approximately −7.84dB. For a two-dimensional metasurface composed of periodically repeated supercells, the far-field radiation can be described, according to array theory, as the product of the element factor and the array factor:

$$\begin{aligned} E_{\mathrm{out}}\left( f_{x},f_{y} \right)={\frac{1}{M}\cdot E}_{\text{sp}}\left( f_{x},f_{y} \right) \cdot\text{A}\text{F}_{\text{super}}\left( f_{x},f_{y} \right),\#\left( S9 \right) \end{aligned}$$

where the prefactor 1/*M* (with *M* = 2 corresponding to the 2×2 mode-region arrangement used in this work) accounts for the amplitude dilution of the principal order caused by the reduced effective illuminated area. $E_{\text{sp}}$ denotes the element factor, which describes the far‑field radiation pattern of a single 8μm mode meta‑pixel and determines its primary diffraction direction. $\text{A}\text{F}_{\text{super}}$ represents the array factor of the nested supercell lattice, which governs the angular side-peak distribution surrounding the target diffraction direction. The factorized form in Eq. (S8) follows the standard array-theory approximation under weak inter-super-pixel coupling since module size is much larger than the wavelength and local periodicity assumptions applied here, which has been widely adopted in metasurface diffraction analysis.

For an ideal infinitely periodic array, the array factor forms Dirac-delta-type diffraction peaks at discrete spatial-frequency locations. The spatial frequencies along the x and y directions can be expressed as:

$$\begin{aligned} f_{x}=\frac{dp}{D_{\text{super}}},\quad f_{y}=\frac{dq}{D_{\text{super}}},\quad\quad p,q\in Z\#\left( S10 \right) \end{aligned}$$

where $D_{\text{super}}$ denotes the nested supercell period, and *dp* and *dq* represent the diffraction-order indices along the x and y directions, respectively.

This indicates that the side peaks are distributed around the target spatial frequency $\left( f_{x0},f_{y0} \right)$ of the individual super-pixel mode, rather than being regenerated around the zero-frequency component or the first-order diffraction angle associated with the enlarged supercell period. For an arbitrary Fourier-coefficient-optimized mode super-pixel, the target diffraction direction can be represented by the corresponding target spatial frequency $\left( f_{x0},f_{y0} \right)$. This spatial frequency is determined by the optimized local phase distribution of the metasurface, and is not necessarily equivalent to the first-order diffraction frequency of a simple grating with period D_._ Under ideal conditions, the far-field main lobe of a single mode super-pixel is centered at $\left( f_{x0},f_{y0} \right)$.

When the mode super-pixel is embedded into a 2×2 nested supercell, the enlarged nested periodicity does not modify the target radiation direction determined by the local mode super-pixel. Instead, the nested supercell introduces additional superlattice diffraction orders around the target spatial frequency through the array factor. Therefore, the diffraction peaks of the complete far-field distribution can be approximately expressed as:

$$\begin{aligned} f_{x}=f_{x0}+\frac{p}{D_{\text{super}}},\#\left( S11 \right) \end{aligned}$$

$$\begin{aligned} f_{y}=f_{y0}+\frac{q}{D_{\text{super}}},\#\left( S12 \right) \end{aligned}$$

where the complete nested supercell period is given by:

$$\begin{aligned} D_{\text{super}}=2ND_{sp}.\#\left( S13 \right) \end{aligned}$$

Here, $D_{\mathrm{sp}}$ is the dimension of a single mode meta‑pixel, and *N* is the repetition count of mode meta‑pixels along the *x* and *y*-directions within each mode region. It can therefore be seen that varying *N* does not change the principal communication direction ($f_{x0}$, $f_{y0,}$), but instead modifies the spatial-frequency spacing between neighboring superlattice side orders distributed around the target peak:

$$\begin{aligned} \Delta f=\frac{1}{D_{\text{super}}}=\frac{1}{2ND_{\mathrm{sp}}}.\#\left( S14 \right) \end{aligned}$$

For a Fourier-coefficient-optimized mode super-pixel, the target radiation direction is determined by the target spatial frequency $\left( f_{x0},f_{y0} \right)$, which originates from the optimized local phase distribution of the 8 μm mode super-pixel rather than from the diffraction frequency of a simple periodic grating. The corresponding propagation angle satisfies the generalized grating relation:

$$\begin{aligned} \sin\theta_{x}=\lambda f_{x0},\sin\theta_{y}=\lambda f_{y0}.\#\left( S15 \right) \end{aligned}$$

The relationship between the angular spacing of adjacent diffraction propagation directions and *N* can be expressed as:

$$\begin{aligned} \Delta\theta\propto\frac{\lambda}{2ND_{\mathrm{sp}}}.\#\left( S16 \right) \end{aligned}$$

As *N* increases, $D_{\text{super}}$becomes larger and *Δf* correspondingly decreases, leading to a reduced angular separation between adjacent side orders. Consequently, if these side orders fall within the angular acceptance window of the target receiver, they can be collected as useful multi-angle signal components. If they fall into non-target receiving channels, they contribute to inter-port crosstalk. Otherwise, if they are not collected by any receiver, they contribute to uncollected optical leakage and system power loss.

When the spatial-frequency sampling density is low, a large portion of the diffracted energy is distributed into side peaks far away from the target direction, thereby reducing the useful collected power at the receiver. Consequently, increasing *N* enhances the spatial-frequency sampling density and allows more side-peak energy to be concentrated within the target reception window.

**Figure S3** compares the relative power distributions of the side peaks for the *N=*1 and *N=*8 configurations. For the *N*=8 case, the main diffraction peak, the first-order side peaks, and the diagonal side peaks associated with the target first-order diffraction mode all fall within the ±1.1∘ reception window, thereby forming nine effective angular channels. **Figure 5** in the main text further presents the far-field diffraction distributions of the four-mode metasurface under single-mode periodic excitation as well as the nested four-mode configurations for *N*=1 and *N*=8.

**Figure S3**. Far-field diffraction-order distributions of the nested four-mode metasurface for (a) *N*=1 and (b) *N*=8. The red dashed line indicates the target diffraction angle, and the shaded region denotes the useful angular window (±1.1°). Compared with *N*=1, the *N*=8 configuration enables both the central peak and multiple side peaks to fall within the useful reception range.

**Section 3: Design Parameter Analysis and Scalability of the NFM Architecture**

The scalability of the nested Fourier metasurface (NFM) can be interpreted from two distinct spatial hierarchies. At the local level, the period and sampling density of each Fourier‑engineered mode super-pixel govern the accessible diffraction momentum space, as well as the precision with which optical power is redistributed among different diffraction orders. At the global level, the number and arrangement of spatially multiplexed mode domains determine the number of pre‑designed addressable mode states and modify the superlattice diffraction spectrum introduced by the nested architecture.

To avoid ambiguity with the repetition number used in the main text, we define the number of nanopillars along one dimension of a mode super-pixel as $N_{p}$, the nanopillar pitch as *P*, and the number of repeated mode super-pixels within each mode region as *N*. The corresponding mode-super-pixel period and mode-region size are

$$\begin{aligned} D_{sp}=N_{p}\times P=8\mu m,\#\left( S17 \right) \end{aligned}$$

and the width of an individual mode region is:

$$\begin{aligned} L_{\mathrm{region}}=D_{\mathrm{super}}=ND_{\mathrm{sp}}.\#\left( S18 \right) \end{aligned}$$

For the configuration considered in this work，$D_{\mathrm{sp}}$= 8μm, $N_{p}$=10, *N* = 8 and $L_{\mathrm{region}}$= 64μm.

The following analysis discusses separately the accessible diffraction orders, the role of discrete Fourier sampling, and the scalability of the spatially multiplexed NFM from the demonstrated 2×2 configuration toward a general *M*×*M* architecture.

**3.1 Accessible Diffraction Orders of the Local Mode Super-Pixel**

According to the grating equation, $\mathrm{Dsp}$ determines the theoretical upper limit of the propagating diffraction orders in the far‑field diffraction. For the simplified case of normal incidence onto a one‑dimensional grating, the grating equation can be simplified as:

$$\begin{aligned} |sin \theta_{m}|=\left| m\frac{\lambda}{\mathrm{Dsp}} \right|\leq1,\#\left（ S19 \right） \end{aligned}$$

where $\theta_{m}$ denotes the propagating diffraction angle, and m corresponds to the diffraction order. For $D_{\mathrm{sp}}$=8μm and λ=1550nm, the maximum number of propagating diffraction orders in the far‑field diffraction for the proposed design framework can be calculated as:

$$\begin{aligned} \left| m \right|\leq\frac{D\mathrm{sp}}{\lambda}=\frac{8\mu m}{1550nm}\approx5.161.\#\left( S20 \right) \end{aligned}$$

Consequently, the propagating diffraction orders for the one‑dimensional case are *m* = 0, ±1, ±2, ±3, ±4, ±5, corresponding to 11 propagating orders within the ideal scalar‑grating description.

For a two‑dimensional metasurface with identical period $\mathrm{Dsp}$ along both orthogonal in‑plane directions, the two‑dimensional grating equations under normal incidence are given by

$$\begin{aligned} \sin\theta_{x}=\frac{m\lambda}{D_{\text{sp}}},\quad\quad\sin\theta_{y}=\frac{n\lambda}{D_{\text{sp}}}.\#\left( S21 \right) \end{aligned}$$

The far‑field propagation constraint for radiative diffraction orders yields

$$\begin{aligned} \sqrt{\sin^{2} \theta_{x}+\sin^{2} \theta_{y}}\leq1.\#\left( S22 \right) \end{aligned}$$

Substituting the grating equations into the propagation condition, we obtain

$$\begin{aligned} \left( \frac{m\lambda}{D_{\text{sp}}} \right)^{2}+\left( \frac{n\lambda}{D_{\text{sp}}} \right)^{2}\leq1.\#\left( S23 \right) \end{aligned}$$

Substituting the numerical parameters $D_{\mathrm{sp}}$=8μm and λ=1550 nm, the inequality further reduces to

$$\begin{aligned} m^{2}+n^{2}\leq\left( \frac{D_{\text{sp}}}{\lambda} \right)^{2}\approx26.63.\#\left( S24 \right) \end{aligned}$$

A total of 89 integer diffraction-order pairs (*m*, *n*), including the zero order, satisfy this ideal free-space propagation condition. Practical utilization of these orders is additionally constrained by the meta-atom response, polarization conversion efficiency, Fourier-sampling degrees of freedom, desired channel isolation, and the acceptance range of the receiver.

Additionally, the phase responses of $N_{p}$ nanopillars inside a meta‑pixel form a discrete spatial sequence. From the Fourier‑synthesis viewpoint, discrete Fourier coefficients govern the complex amplitudes of different diffraction components, and the number of nanopillars sets the degrees of freedom for discrete sampling to approximate the target continuous Fourier phase profile. Increasing $N_{p}$ can reduce phase‑sampling and spatial quantization errors and provide more degrees of freedom for light‑energy redistribution among diffraction orders. Nevertheless, the distinct roles of $N_{p}$ and $D_{\text{sp}}$ should be clarified: the accessible far‑field propagating diffraction range is fundamentally determined by $D_{\text{sp}}/\lambda$, while the synthesis accuracy of diffraction coefficients relies on the sampling‑unit count $N_{p}$. Following $D_{\mathrm{sp}}$= $N_{p}$ × *P*, these two parameters are coupled for a fixed nanopillar period P. Raising $N_{p}$ under such condition enlarges the meta‑pixel period and alters the range of available diffraction orders. Accordingly, to investigate Fourier‑sampling convergence, one should tune the sampling density with $D_{\mathrm{sp}}$ fixed, or separate the influence induced by $D_{\mathrm{sp}}$ variation from that originating from larger $N_{p}$. Numerical results demonstrate that the efficiencies of target diffraction orders converge under sufficiently dense spatial sampling. Further increasing the sampling count beyond this convergence regime brings only marginal performance improvement, accompanied by more meta‑atoms and higher design‑and‑fabrication complexity.

**3.2 Spatial-Mode Scalability of the NFM Architecture from 2×2 to *M*×*M***

The NFM demonstrated in this work employs a 2×2 arrangement containing four independently designed mode regions. The same concept can, in principle, be generalized to an *M*×*M* arrangement containing up to *M*^2^ pre-designed spatially addressable mode regions. Thus, a 3×3, 4×4, or 5×5 architecture could accommodate up to 9, 16, or 25 local mode regions, respectively. These numbers describe the size of the available spatial mode library. The number of modes simultaneously activated or routed remains dependent on the applied optical-addressing pattern and the intended system configuration.

When the physical size of each mode region $L_{region}$, is kept unchanged, increasing M enlarges the nested-supercell period according to

$$\begin{aligned} D_{super}=ML_{region}.\#\left( S25 \right) \end{aligned}$$

As described by the superlattice diffraction model in Section 2, the corresponding spatial-frequency spacing scales as 1/$D_{super}$. Around the target diffraction direction, the neighboring angular spacing can therefore be approximated as:

$$\begin{aligned} \Delta\theta\approx\frac{\lambda}{ML_{\mathrm{region}}}.\#\left( S26 \right) \end{aligned}$$

Based on the above principles, we calculate the angular separations of the on‑axis first‑ and second‑order diffraction orders for different values of *M*, and evaluate the useful energy gain collected within the ±1.1° receiving window. The detailed data are summarized in **Table S1**. Increasing *M* enlarges the library of available spatial modes and generates denser clusters of superlattice diffraction components around each locally designed target direction. According to the aperture analysis presented in **Section 2**, the reduced spatial duty ratio allows more adjacent superlattice orders to fall within the central diffraction envelope.

For a periodic structure, the Fourier coefficients of the aperture function scale inversely with the supercell period. Therefore, the absolute field amplitude of the principal order (0,0) is diluted by a factor of 1/*M* relative to the isolated mode super-pixel:

$$\begin{aligned} E_{\text{main}}\left( M \right)=\frac{1}{M}\cdot E_{\text{sp}}.\#\left( S27 \right) \end{aligned}$$

Consequently, the absolute efficiency of the principal order is:

$$\begin{aligned} \eta_{\text{main}}\left( M \right)=\frac{\eta_{\text{iso}}}{M^{2}},\#\left( S28 \right) \end{aligned}$$

where $\eta_{\text{iso}}$ denotes the absolute diffraction efficiency of a mode region with *N*=8 mode super‑pixels. This 1/*M*^2^ scaling is a direct consequence of energy conservation: as *M* increases, the same incident power is distributed over a denser grid of superlattice orders.

To further investigate the diffraction of discrete superlattice orders, the electric field of each far‑field diffraction order $\left( dp,dq \right)$-th superlattice can be expressed as:

$$\begin{aligned} E_{pq}\left( M \right)=\frac{1}{M}\cdot E_{\text{super}\text{-}\text{pixel}}\cdot\text{sinc}\left( \frac{dp}{M} \right)\cdot\text{sinc}\left( \frac{dq}{M} \right),\#\left( S29 \right) \end{aligned}$$

and the corresponding absolute efficiency is:

$$\begin{aligned} \eta_{pq}\left( M \right)=\frac{\eta_{\text{iso}}}{M^{2}}\cdot\text{sinc}^{2}\left( \frac{dp}{M} \right)\cdot\text{sinc}^{2}\left( \frac{dq}{M} \right).\#\left( S30 \right) \end{aligned}$$

For a receiver with an angular acceptance window Ω (where Ω denotes the set of all diffraction orders within the ±1.1^∘^ window), the total collected power is the sum of the absolute efficiencies of all diffraction orders falling within the window:

$$\begin{aligned} \eta_{\text{window}}\left( M \right)=\sum_{\left( dp,dq \right)\in\Omega} \eta_{pq}\left( M \right)=\frac{\eta_{\text{iso}}}{M^{2}}\sum_{\left( dp,dq \right)\in\Omega} \text{sinc}^{2}\left( \frac{dp}{M} \right)\text{sinc}^{2}\left( \frac{dq}{M} \right).\#\left( S31 \right) \end{aligned}$$

The useful power gain is the ratio of the window‑collected power to the principal‑order power alone and is given by:

$$\begin{aligned} G_{\text{win}}\left( M \right)=\frac{\eta_{\text{window}}\left( M \right)}{\eta_{\text{main}}\left( M \right)}=\sum_{\left( dp, dq \right)\in\Omega} \mathrm{sinc}^{2} \left( \frac{dp}{M} \right)\mathrm{sinc}^{2} \left( \frac{dq}{M} \right).\#\left( S32 \right) \end{aligned}$$

In decibels:

$$\begin{aligned} G_{\text{win}}\left( M \right)=10\log_{10} \left[ \sum_{\left( dp,dq \right)\in\Omega} \mathrm{sinc}^{2} \left( \frac{dp}{M} \right)\mathrm{sinc}^{2} \left( \frac{dq}{M} \right) \right].\#\left( S33 \right) \end{aligned}$$

For the representative ±1.1° window and the 2×2 arrangement (*M*=2), the window contains *dp, dq* ∈ {−1, 0, 1}, comprising 9 orders in total:

$$G_{\text{win}}\left( 2 \right)=10\log_{10} \left[ 1+4\times\text{sinc}^{2}\left( 0.5 \right)+4\times\text{sinc}^{2}\left( 0.5 \right)\text{sinc}^{2}\left( 0.5 \right) \right]$$

$$=10\log_{10} \left( 3.278 \right)\approx5.16 \text{dB}$$

For the 3×3 arrangement (*M*=3), the window contains *dp, dq* ∈ {-2, −1, 0, 1, 2}, comprising 25 orders in total:

$$G_{\text{win}}\left( 3 \right)=10\log_{10} \left[ 1+4\times sinc^{2}\left( 1/3 \right)+4\times sinc^{2}\left( 2/3 \right)+4\times sinc^{2}\left( 1/3 \right)sinc^{2}\left( 1/3 \right)+8\times sinc^{2}\left( 2/3 \right)sinc^{2}\left( 1/3 \right)+4\times sinc^{2}\left( 2/3 \right) \right]=10\log_{10} \left( 7.343 \right)\approx8.66 \text{dB}$$

For the 4×4 arrangement (*M*=4), the window contains *dp, dq* ∈ {-3, -2, −1, 0, 1, 2, 3}, comprising 49 orders in total:

$$G_{\text{win}}\left( 4 \right)=10\log_{10} \left[ 1+4\times sinc^{2}\left( 0.25 \right)+4\times sinc^{2}\left( 0.5 \right)+4\times sinc^{2}\left( 0.75 \right)+4\times sinc^{2}\left( 0.25 \right)sinc^{2}\left( 0.25 \right)+4\times sinc^{2}\left( 0.5 \right)sinc^{2}\left( 0.5 \right)+4\times sinc^{2}\left( 0.75 \right)sinc^{2}\left( 0.75 \right)+8\times sinc^{2}\left( 0.25 \right)sinc^{2}\left( 0..5 \right)+8\times sinc^{2}\left( 0.25 \right)sinc^{2}\left( 0.75 \right)+8\times sinc^{2}\left( 0.5 \right)sinc^{2}\left( 0.75 \right) \right]=10\log_{10} \left( 13.045 \right)\approx11.15 \text{dB }$$

For the 5×5 arrangement (*M*=5), the window contains *dp, dq* ∈ {-4, -3, -2, −1, 0, 1, 2, 3, 4}, comprising 81 orders in total:

$$G_{\text{win}}\left( 5 \right)=10\log_{10} \left[ 1+4\times sinc^{2}\left( 0.2 \right)+4\times sinc^{2}\left( 0.4 \right)+4\times sinc^{2}\left( 0.6 \right)+4\times sinc^{2}\left( 0.8 \right)+4\times sinc^{2}\left( 0.2 \right)sinc^{2}\left( 0.2 \right)+4\times sinc^{2}\left( 0.4 \right)sinc^{2}\left( 0.4 \right)+4\times sinc^{2}\left( 0.6 \right)sinc^{2}\left( 0.6 \right)+4\times sinc^{2}\left( 0.8 \right)sinc^{2}\left( 0.8 \right)+8\times sinc^{2}\left( 0.2 \right)sinc^{2}\left( 0.4 \right)+8\times sinc^{2}\left( 0.2 \right)sinc^{2}\left( 0.6 \right)+8\times sinc^{2}\left( 0.2 \right)sinc^{2}\left( 0.8 \right)+8\times sinc^{2}\left( 0.4 \right)sinc^{2}\left( 0.6 \right)+8\times sinc^{2}\left( 0.4 \right)sinc^{2}\left( 0.8 \right)+8\times sinc^{2}\left( 0.6 \right)sinc^{2}\left( 0.8 \right) \right]=10\log_{10} \left( 20.380 \right)\approx13.09 \text{d}B$$

The window collection efficiency can also be expressed in terms of the main-order efficiency and the useful power gain:

$$\begin{aligned} \eta_{\text{window}}\left( M \right)=\eta_{\text{main}}\left( M \right)\cdot{10}^{G_{\text{win}}\left( M \right)/10}\#\left( S34 \right) \end{aligned}$$

or equivalently, in terms of the isolated region efficiency:

$$\begin{aligned} \eta_{\text{window}}\left( M \right)=\frac{\eta_{\text{iso}}}{M^{2}}\cdot{10}^{G_{\text{win}}\left( M \right)/10}\#\left( S35 \right) \end{aligned}$$

The results in **Table S1** reveal two complementary effects: as *M* increases, the principal-order efficiency decreases as 1/*M*^2^, while the number of superlattice orders within the ±1.1^∘^ window increases from 9 to 81. The power of the principal order is thus redistributed among the denser superlattice orders, compensating for the dilution and yielding a nearly constant window collection efficiency of approximately 81.5% of the isolated region efficiency across all configurations. The useful power gain $G_{\text{win}}\left( M \right)$ increases monotonically with *M* (from 5.16 dB to 13.09 dB), but with diminishing returns as higher-order sidebands contribute progressively less power. This verifies that the NFM architecture redistributes optical power rather than generating additional power or dissipating it, which forms the core mechanism supporting its scalability. Scalability can be pursued along two complementary routes: incorporating additional diffraction orders from individual mode super-pixels, or expanding the spatial multiplexing array beyond 2×2. These routes can also be combined. However, larger *M* reduces angular spacing between neighboring superlattice components, improving energy capture within the window but imposing stricter requirements on receiver resolution, optical alignment, and addressing field of view. The 2×2 configuration is therefore a proof-of-concept demonstration, not a fundamental limit; larger arrays remain compatible with the same NFM design principles, with practical bounds determined by mode count, power budget, crosstalk tolerance, receiver resolution, and addressing capability.

**Table S1**. Angular spacing and receiver window gain for different *M*×*M* mode-region arrangement

| **Arrangement** | | **2×2** | **3×3** | **4×4** | **5×5** |
| --- | --- | --- | --- | --- | --- |
| Addressable mode regions | | 4 | 9 | 16 | 25 |
| $D_{\mathrm{super}}$ | | 128μm | 192μm | 256μm | 320μm |
| First-order angular spacing Δθ | 0.694° | | 0.463° | 0.347° | 0.278° |
| Second-order angular spacing 2Δθ | | 1.388° | 0.926° | 0.694° | 0.556° |
| Orders within ±1.1° window | | 9 | 25 | 49 | 81 |
| Useful Power Gain | | 5.16 dB | 8.66 dB | 11.15 dB | 13.09 dB |
| Addressable mode regions | | 4 | 9 | 16 | 25 |
| $\eta_{\text{main}}$=1/M^2^ (relative, $\eta_{\text{iso}}$=1) | | 0.2500 | 0.1111 | 0.0625 | 0.0400 |
| $\eta_{\text{window}}\left( M \right)=\eta_{\text{main}}\left( M \right)\cdot{10}^{G_{\text{win}}\left( M \right)/10}$ | | 0.8200 | 0.8158 | 0.8156 | 0.8152 |

**Figure S3.** Near-field electric-field distributions of the NFM supercell under DMD-activated illumination of the four mode regions. Panels (a)–(d) correspond to selective illumination of Modes 1–4 regions, respectively.

**Figure S4.** FDTD-simulated far-field diffraction patterns of the nested Fourier metasurface under the *N*=8 configuration. Panels (a)–(d) show the simulated far-field distributions when each mode region is individually activated by DMD illumination at 1550 nm.

**Section 4: Multipath Delay Channel Modeling and Communication Performance Analysis of the Nested-Supercell Architecture**

Due to the adoption of the *N*=8 mode super-pixel arrangement, in addition to the target principal diffraction peak, eight neighboring side peaks can also fall within the effective angular acceptance window of the receiver for the first-order diffraction channel. Consequently, nine effective angular propagation channels are formed around the target communication direction. Although these diffraction orders are clustered near the same target direction, their propagation angles still exhibit slight differences, leading to different free-space propagation paths and corresponding propagation delays.

To evaluate the influence of this angular multipath effect on system performance, we further establish a free-space multipath delay-channel model for the *N*=8 nested-supercell architecture and investigate its impact on communication performance metrics such as the bit-error rate (BER). For a diffraction order corresponding to spatial frequency $\left( f_{x},f_{y} \right)$, the free-space propagation direction cosines satisfy:

$$\begin{aligned} s_{x}=\lambda f_{x},s_{y}=\lambda f_{y},s_{z}=\sqrt{1-s_{x}^{2}-s_{y}^{2}},\#S\left( 36 \right) \end{aligned}$$

where

$$\begin{aligned} s_{z}=\cos\theta.\#\left( S37 \right) \end{aligned}$$

Assuming a free-space propagation distance *L*, the corresponding optical propagation path length is approximately given by:

$$\begin{aligned} \tau\left( f_{x},f_{y} \right)=\frac{L}{c \cos\theta}=\frac{L}{c\sqrt{1-\left( \lambda f_{x} \right)^{2}-\left( \lambda f_{y} \right)^{2}}},\#\left( S38 \right) \end{aligned}$$

where $c$ is the speed of light in vacuum. The relative delay of the i-th multipath component with respect to the target principal diffraction peak $\left( f_{x0},f_{y0} \right)$ is given by:

$$\begin{aligned} \Delta\tau_{i}=\tau\left( f_{xi},f_{yi} \right)-\tau\left( f_{x0},f_{y0} \right).\#\left( S39 \right) \end{aligned}$$

Therefore, the clustered angular channels generated by the proposed metasurface can be physically interpreted as a set of spatial multipath components with different propagation delays. The maximum delay spread and the root-mean-square (RMS) delay spread of the useful collected components are respectively defined as:

$$\begin{aligned} \Delta\tau_{\text{max}}=\max_{i} \{\Delta\tau_{i}\}-\min_{i} \{\Delta\tau_{i}\},\tau_{\text{rms}}=\sqrt{\frac{\sum_{i} P_{i}\left( \Delta\tau_{i}-\overline{\Delta\tau} \right)^{2}}{\sum_{i} P_{i}}}.\#\left( S40 \right) \end{aligned}$$

The mean delay is defined as:

$$\begin{aligned} \overline{\Delta\tau}=\frac{\sum_{i} P_{i}\Delta\tau_{i}}{\sum_{i} P_{i}}.\#\left( S41 \right) \end{aligned}$$

For the proposed *N*=8 nested-supercell configuration, the calculated maximum delay spread under a propagation distance of *L*=5 cm is $\Delta\tau_{\text{max}}\approx0.84\text{ps}$，which is significantly smaller than the bit duration $T_{b}=20\text{ps}$ of a 50Gbps NRZ-OOK system. This result indicates that although the proposed nested supercell forms multiple angular propagation channels, the resulting delay spread remains much smaller than the symbol period. Therefore, the induced temporal dispersion is relatively weak and introduces only limited inter-symbol interference (ISI).

To further analyze the influence of the nested supercell on the optical communication link, the received signal can be modeled using a conventional tapped-delay-line (TDL) multipath channel model:

$$\begin{aligned} y\left( t \right)=\sum_{i=1}^{K} a_{i} x\left( t-\Delta\tau_{i} \right),\#\left( S42 \right) \end{aligned}$$

where $x\left( t \right)$ denotes the transmitted NRZ-OOK signal, $a_{i}$ represents the amplitude weight of the i-th angular channel, and K is the number of useful angular propagation channels (*K*=9 for the *N*=8 configuration in the 2×2 mode-region arrangement used in this work). The original amplitude weight of each path is determined from the corresponding diffraction power as:

$$\begin{aligned} a_{i}=\frac{\sqrt{P_{i}}}{\sum_{j=1}^{K} \sqrt{P_{j}}}.\#\left( S43 \right) \end{aligned}$$

For the *N*=8 configuration, the useful relative power collected from the first-order diffraction channel can be calculated as:

$$\begin{aligned} p_{\text{useful}}=\sum_{i=1}^{9} P_{i}==1+4\times0.4053+4\times0.1643=1+1.6211+0.6570=3.2782\#\left( S44 \right) \end{aligned}$$

Therefore, the total received amplitude remains identical for different channel configurations, ensuring that the BER variation originates entirely from the ISI induced by nonzero delay spread, rather than from additional received-power enhancement. On the other hand, if the spatial energy-concentration effect introduced by the proposed nested supercell is taken into account, the unnormalized amplitudes can be directly adopted. In this case, the effective power gain of the *N*=8 configuration relative to the *N*=1 configuration can be expressed as:

$$\begin{aligned} G_{\text{power}}=10\log_{10} \left( \sum_{i=1}^{K} P_{i} \right)\approx+5.16 \text{dB}\text{.}\#\left( S45 \right) \end{aligned}$$

**Table 1** in the main text summarizes the angular-channel characteristics of the nested four-mode metasurface architecture for all four modes. The results indicate that the proposed nested supercell not only forms clustered angular channels, but also enhances useful energy collection around the target direction through multi-angle joint reception.

It should be noted that the impact of the angular multipath effect strongly depends on the transmission scenario. In chip-scale optical interconnects, free-space optical switching, and short-reach free-space optical interconnect systems, the propagation distance is typically on the order of centimeters to decimeters. Consequently, the optical path differences among different angular channels remain relatively small, leading to delay spreads generally ranging from sub-picosecond to several picoseconds, which are still far below the symbol duration of high-speed optical communication systems. Therefore, the temporal dispersion induced by angular multipath propagation introduces only limited inter-symbol interference (ISI) and BER degradation, while the overall system performance remains primarily determined by received optical power, diffraction efficiency, and noise level.

Overall, the above analysis shows that increasing the nested-supercell dimension *N* enables the formation of denser angular side peaks around the target diffraction direction while preserving the principal communication direction. These side peaks collectively form angular multipath channels within the receiver acceptance window and introduce sub-picosecond-scale delay spread. For the 50Gbps, 5cm free-space link investigated in this work, the resulting delay spread remains much smaller than the system bit period, leading to only minor BER degradation while simultaneously providing additional spatial energy-concentration gain.

**Section 5: System-Level Modeling of Metasurface-Enabled Free-Space Optical Channels**

This part provides the free-space optical communications model in details. The communication system consists of an ideal optical transmitter, the optically addressed metasurface switching module, and a simplified receiver model, where the transmitted signal can be expressed as:

$$\begin{aligned} \text{s}\text{(}\text{t}\text{)}\text{ }\text{=}\text{ }\text{A}\text{(}\text{t}\text{)}\cos\text{(}\text{ωt}\text{ }\text{+}\text{ }\text{ϕ}\text{)}\text{,}\#\text{(}\text{S46}\text{)} \end{aligned}$$

where A(*t*) is modulation signal and *ω* is the carrier frequency. For NRZ-OOK modulation, the signal envelope can be expressed as:

$$\begin{aligned} \text{A(}\text{t}\text{)}\text{ }\text{=}\sum_{\text{k}} \text{a}_{\text{k}}\text{p}\text{(}\text{t-kT}\text{)}\text{.}\#\text{(}\text{S47}\text{)} \end{aligned}$$

where $\text{a}_{\text{k}}$ ∈{0,1}, $\text{p}\text{(}\text{t}\text{)}$ is the pulse shape and *T* bit period.

In channel modeling, the metasurface serves as a spatial router, mapping distinct diffraction orders to independent communication channels. The output signal of each channel is expressed as:

$$\begin{aligned} \text{y}_{\text{i}}\text{(}\text{t}\text{)}\text{ }\text{=}\text{ }\text{h}_{\text{i}}\text{(}\text{t}\text{)}\text{ }\text{∗}\text{ }\text{x}\text{(}\text{t}\text{)}\text{ }\text{+}\text{ }\text{n}\text{(}\text{t}\text{)}\text{,}\#\text{(}\text{S48}\text{)} \end{aligned}$$

where $\text{h}_{\text{i}}\text{(}\text{t}\text{)}$ is the channel impulse response and $\text{n}\text{(}\text{t}\text{)}$ is noise. Each diffraction mode corresponds to a different channel:

$$\begin{aligned} \text{h}_{\text{i}}\text{(}\text{t}\text{)}\text{ }\text{=}\text{ }\text{η}_{\text{i}}\text{δ}\text{(}\text{t}\text{)}\text{.}\#\text{(}\text{S49}\text{)} \end{aligned}$$

Where $\text{η}_{\text{i}}$ is the diffraction efficiency.

Inter-port crosstalk is defined as:

$$\begin{aligned} \text{X}\text{T}_{\text{i}}\text{ }\text{=}\text{ }\text{10}\text{log}_{\text{10}} \left( \frac{\text{P}_{\text{i}}}{\sum_{\text{j}\text{≠i}} \text{P}_{\text{j}}} \right),\#(S50) \end{aligned}$$

where $\text{P}_{\text{i}}$ is the desired port and $\text{P}_{\text{j}}$ is the interference ports.

The received signal is defined as:

$$\begin{aligned} \text{r}\text{(}\text{t}\text{)}\text{ }\text{=}\sum_{\text{k}} \text{a}_{\text{k}}\text{p(}\text{t-kT}\text{)}\text{ }\text{+}\text{ }\text{n}\text{(}\text{t}\text{)}\text{.}\#\text{(}\text{S51}\text{)} \end{aligned}$$

Eye diagram quality depends on insertion loss, crosstalk and noise level.

System performance is quantified by the bit error rate (BER), defined as:

$$\begin{aligned} \text{BER}\text{ }\text{=}\text{ }\frac{\text{1}}{\text{2}}\text{erfc}\left( \frac{\text{SNR}}{\sqrt{\text{2}}} \right),\#(S52) \end{aligned}$$

where

$$\begin{aligned} \text{SNR}\text{ }\text{=}\text{ }\frac{\text{P}_{\text{signal}}}{\text{P}_{\text{noise}}}.\#(S53) \end{aligned}$$

To realistically evaluate the intrinsic physical-channel performance of the metasurface, the system model incorporates several non-ideal factors. Specifically, insertion loss and inter-port crosstalk are extracted from full-wave FDTD simulations, while additive white Gaussian noise (AWGN) is introduced at the receiver. In addition, a first-order low-pass filter with a 3-dB bandwidth of 18 GHz is employed to emulate the finite receiver bandwidth. The noise is modeled as a normalized Gaussian distribution with a standard deviation of *σ*=0.03-0.04, which is typical for optical communication channels.

No digital signal processing (DSP) techniques, including feed-forward equalization, adaptive equalization, or advanced forward-error-correction (FEC) encoding and decoding, are adopted in the performance evaluation. Therefore, the reported BER represents the pre-FEC raw performance and directly reflects the intrinsic impairments induced by insertion loss, inter-channel crosstalk, and bandwidth limitation. A typical hard-decision FEC (HD-FEC) BER threshold of 10^-3^, which is widely applied in optical interconnect systems, is used as the evaluation criterion. In practical implementations, enhanced FEC schemes such as soft-decision FEC can be utilized to further reduce the error rate and improve system robustness.
